# Structural basis of DNA binding by the WhiB-like transcription factor WhiB3 in *Mycobacterium tuberculosis*

**DOI:** 10.1016/j.jbc.2023.104777

**Published:** 2023-05-02

**Authors:** Tao Wan, Magdaléna Horová, Vimmy Khetrapal, Shanren Li, Camden Jones, Andrew Schacht, Xinghui Sun, LiMei Zhang

**Affiliations:** 1 Department of Biochemistry, University of Nebraska-Lincoln, Lincoln, Nebraska, USA; 2 Redox Biology Center, University of Nebraska-Lincoln, Lincoln, Nebraska, USA; 3 Nebraska Center for Integrated Biomolecular Communication, University of Nebraska-Lincoln, Lincoln, Nebraska, USA

**Keywords:** Wbl family, WhiB3, transcription factor, iron–sulfur cluster, X-ray crystallography

## Abstract

*Mycobacterium tuberculosis* (*Mtb*) WhiB3 is an iron–sulfur cluster-containing transcription factor belonging to a subclass of the WhiB-Like (Wbl) family that is widely distributed in the phylum Actinobacteria. WhiB3 plays a crucial role in the survival and pathogenesis of *Mtb*. It binds to the conserved region 4 of the principal sigma factor (σ^A^_4_) in the RNA polymerase holoenzyme to regulate gene expression like other known Wbl proteins in *Mtb*. However, the structural basis of how WhiB3 coordinates with σ^A^_4_ to bind DNA and regulate transcription is unclear. Here we determined crystal structures of the WhiB3:σ^A^_4_ complex without and with DNA at 1.5 Å and 2.45 Å, respectively, to elucidate how WhiB3 interacts with DNA to regulate gene expression. These structures reveal that the WhiB3:σ^A^_4_ complex shares a molecular interface similar to other structurally characterized Wbl proteins and also possesses a subclass-specific Arg-rich DNA-binding motif. We demonstrate that this newly defined Arg-rich motif is required for WhiB3 binding to DNA *in vitro* and transcriptional regulation *in Mycobacterium smegmatis*. Together, our study provides empirical evidence of how WhiB3 regulates gene expression in *Mtb* by partnering with σ^A^_4_ and engaging with DNA *via* the subclass-specific structural motif, distinct from the modes of DNA interaction by WhiB1 and WhiB7.

The causative agent of tuberculosis, *Mycobacterium tuberculosis (Mtb)*, continues to affect millions of people annually with high mortality, and the devastation caused by this pathogen is exacerbated by the ongoing COVID-19 and HIV epidemic (1, 2). The survival and persistence of *Mtb* in the host depends on a complex regulatory system to rapidly sense and respond to various assaults launched by the host immune system, such as acidic, oxidative, and nutritional stress. The seven members of WhiB-like (Wbl) family proteins found in *Mtb,* namely WhiB1-7, are key components in this regulatory system. Wbl proteins are a group of small iron–sulfur cluster ([4Fe-4S])-bound proteins first discovered in *Streptomyces* and exclusive to Actinobacteria (3, 4). Members of Wbl proteins play versatile and nonredundant roles in regulating biological processes and responding to various stresses *Mtb* encounters in the host, such as oxidative stress (WhiB1-7), cell division (WhiB2), acidic stress and nutritional starvation (WhiB3), virulence and reactivation (WhiB3, WhiB5, and WhiB6), and antibiotic resistance (WhiB2 and WhiB7) (5, 6, 7, 8). Among them, *Mtb* WhiB3 is one of the key global regulators involved in the early-stage response to acidic stress inside host macrophages (9, 10). It is exploited by *Mtb* to maintain redox and metabolic homeostasis in response to various host-generated redox stress, acidic stress, and carbon starvation and induced by hypoxia and nitric oxide *in vitro* (10, 11, 12, 13, 14, 15, 16, 17, 18). In *Streptomyces*, the WhiB3 ortholog, WhiD, is required for the late stage of sporulation (19, 20).

Correlated with their diverse roles in regulating gene expression, *Mtb* Wbl proteins share only 30 to 50% sequence identity and represent five different subclasses of Wbl proteins widely distributed in Actinobacteria. Two conserved motifs are found in all Wbl proteins—a [4Fe-4S]-cluster binding domain containing four conserved Cys and a “**G**[I/V]**W[**G/A]**G**” motif (the invariant residues are highlighted in bold fonts and the preferred residues are underlined; the same notations are used below), which is also referred to as the β turn (Fig. S1). The C terminus of Wbl proteins has been implicated in DNA binding since many Wbl proteins feature a cluster of basic residues in this region that is predicted to be in a helix-turn-helix fold (4). However, among the Wbl proteins in *Mtb*, only WhiB7 has a defined DNA-binding motif, the AT-hook (“**RGRP**”), in the C terminus. WhiB7 AT-hook preferably binds to the AT-rich sequence upstream of the −35 element in the target promoters. WhiB3 was also suggested to possess a C-terminal AT-hook–like motif (13). But these basic residues in the C-terminal WhiB3 are not conserved in the WhiB3 subclass (Fig. S1), and their role in WhiB3 binding to DNA has not been verified. Even less known is the N terminus of Wbl proteins prior to the Fe–S cluster–binding motif. This region varies substantially in length and sequence among the Wbl proteins (Fig. S1) and lacks information regarding its significance to the function of Wbl proteins.

Several *Mtb* Wbl proteins, including WhiB1, WhiB3, and WhiB7, have been shown to regulate gene expression in the [4Fe-4S]-bound (holo-) form by binding to the conserved region 4 in the σ^70^-family principal sigma factor σ^A^ (σ^A^_4_) in the RNA polymerase (RNAP) holoenzyme (18, 21, 22). Other *Mtb* Wbl proteins except WhiB5 have also been reported to bind to σ^A^_4_ in a [4Fe-4S]-dependent manner (23). Moreover, a recent study has shown that the WhiB3 ortholog of *Streptomyces venezuelae* also binds to region 4 of the principal sigma factor σ^HrdB^, suggesting a shared mechanism of action by Wbl proteins in Actinobacteria (24).

Recent advances in the structural and biochemical characterization of the Wbl proteins have shed light on how Wbl proteins partner with σ^A^ to regulate gene expression. *Mtb* WhiB1 is the first Wbl protein that has been structurally characterized at the atomic level, first in the free holo-form by nuclear magnetic resonance and subsequently in the σ^A^_4_-bound form by X-ray crystallography (22, 25). Together with the molecular and biochemical analyses, these studies reveal an unexpected molecular interface in the WhiB1:σ^A^_4_ complex dominated by hydrophobic interactions and support a new molecular mechanism of transcription regulation by WhiB1 in Actinobacteria (25). Subsequently, the crystal structure of WhiB7 in complex with σ^A^_4_ and its own promoter (*P*_*whiB7*_) and the single-particle cryo-electron microscopy (cryo-EM) structure of WhiB7 bond to σ^A^ in RNAP and *P*_*whiB7*_ were reported by our group and by the Campbell group, respectively (26, 27). The WhiB7 AT-hook binding site has the characteristics of A-track DNA (*i.e.*, a short run of consecutive four or more adenine-thymine base pairs), which possesses distinct structural properties from canonical B-form DNA, including narrow minor grooves, high propeller twists, and DNA bending toward the AT-rich minor groove (see the review in (28)). Analysis of the 3D structures reveals the structural basis for how WhiB7 binding to the AT-rich region opens the minor groove, reversely bends the DNA in the direction opposite to the expected intrinsic bending of A-track DNAs, and orchestrates with σ^A^ for transcriptional regulation. Together, these studies provide an atomic view of how WhiB7 activates gene expression by coordinating DNA binding with σ^A^_4_ *via* its AT-hook and unravel the WhiB7 subclass-specific structural features that enable WhiB7 to function differently from WhiB1 (26, 27).

WhiB3 is one of the most extensively investigated *Mtb* Wbl proteins owing to its importance for the pathogenesis of *Mtb* (10, 11, 12, 13, 14, 15, 16, 17, 18, 29). However, neither the DNA-binding motif nor the DNA-binding preference of WhiB3 has been determined to date. It remains enigmatic how WhiB3 binds to σ^A^_4_ and DNA for transcriptional regulation. Here, we report crystal structures of the σ^A^_4_-bound WhiB3 alone and in complex with DNA at 1.5 Å and 2.45 Å, respectively. Together, the results from our structural, biochemical, and functional analyses uncover an essential DNA-binding motif in WhiB3 and shed light on how WhiB3 coordinates with σ^A^_4_ and interacts with DNA for transcriptional regulation. By structural comparison, we provide insights into how WhiB3 functions differently from WhiB1 and WhiB7 in *Mtb* by binding to the same site on σ^A^_4_ and utilizing the subclass-specific structural motif for DNA binding.

## Results

### Crystal structure of the WhiB3:σ^A^_4_-βtip complex

**Figure 1:**
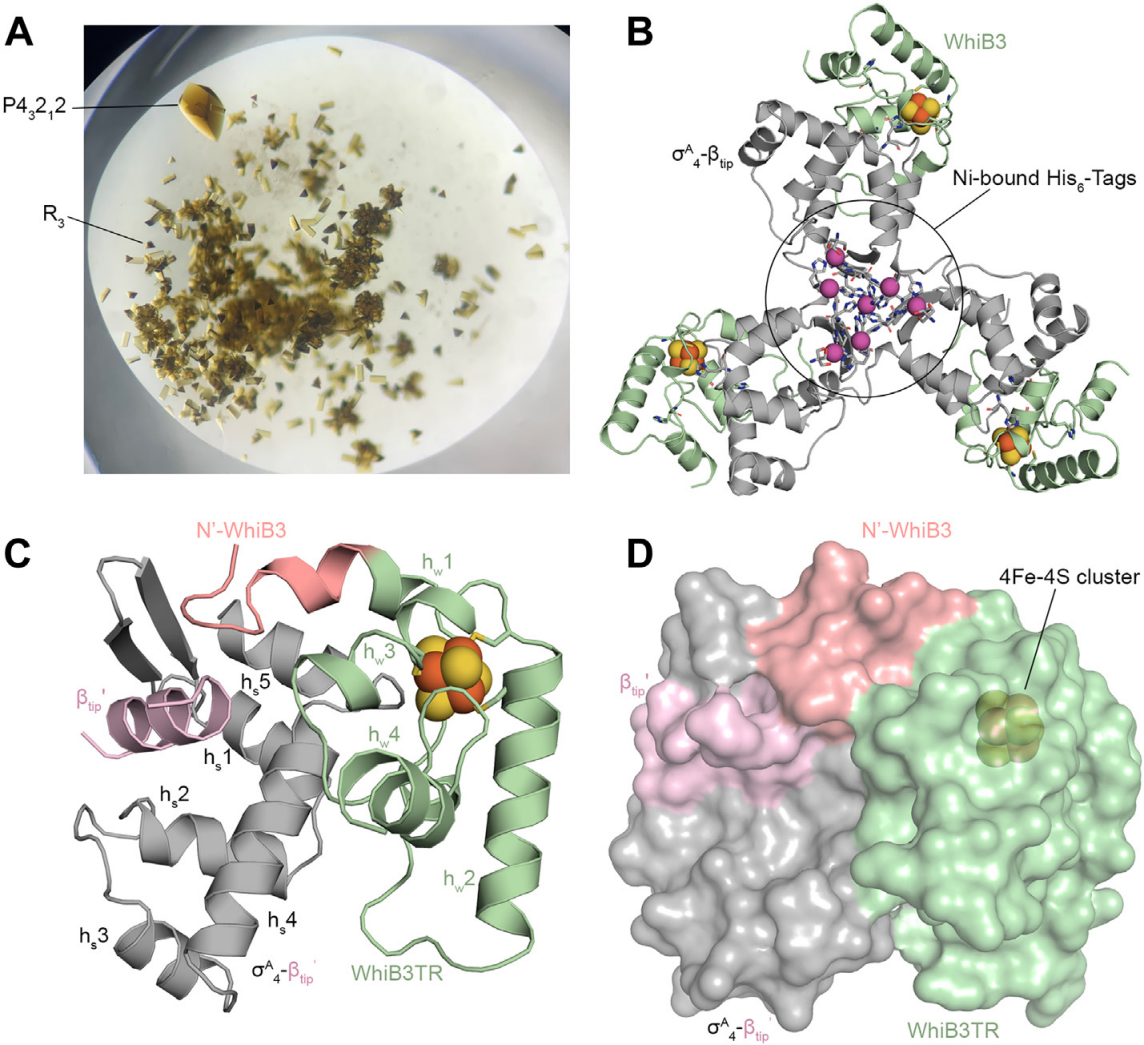
**Overview of the WhiB3TR:σ**^**A**^_**4-**_**β**_**-tip**_**structure.***A*, an optical image of the WhiB3TR:σ^A^_4_-β_tip_ crystals. The two forms of crystals, P4_3_2_1_2 and R3, respectively, are shown in the same crystal drop. *B*, the trimeric structure of the WhiB3TR:σ^A^_4_-β_tip_ complexes in the R3 form. The trimer is centered on the His_6_-tags in the N terminus of σ^A^_4-_β_tip_, which bind to Ni ions from the crystallization solution. The side chains of the His_6_-tag residues are shown in *sticks,* and the Ni atoms are shown in *magenta spheres*. *C*, cartoon representation of the WhiB3:σ^A^_4_-β_tip_^ʹ^ complex in the R3 form, in which β_-tip_ from the neighboring complex molecule (β_tip_^ʹ^) is shown to reflect its interaction with σ^A^_4_ and WhiB3. The four helices in WhiB3 and five helices in σ^A^_4_ are labeled as h_w_1-4 and h_s_1-5, respectively. *D*, surface representation of the WhiB3TR: σ^A^_4_-β_tip_ complex, demonstrating the enclosed [4Fe-4S] cluster binding pocket in the complex. In all the structures, C atoms of WhiB3 are colored *pale green* with the N-terminus residues (aa 6–16) in *salmon pink*, σ^A^_4_ in *gray*, and β_tip_ in *pink*. The [4Fe-4S] clusters are shown in *spheres*, with Fe colored *orange* and S in *yellow*. [4Fe-4S] cluster, iron–sulfur cluster containing four iron and four sulfur ions; σ^A^_4_-β_tip_, σ^A^_4_ fused with β_tip_ by an artificial linker; β_tip,_ the C-terminal flap tip helix of the RNAP β-subunit; His6-tag, hexa histidine-tag; WhiB3TR, a truncated WhiB3 without C-terminal loop region (aa 91–102).

As described in the previous studies, a chimeric protein denoted σ^A^_4_-β_tip_ was used for the crystallographic characterization of the σ^A^_4_-bound WhiB3 by fusing σ^A^_4_ with the RNAP β-subunit flap tip helix (β_tip_) *via* an artificial linker to mimic the interaction between σ^A^ and the β subunit in the RNAP holoenzyme (see Experimental procedures) (26, 30, 31). We also generated a truncated WhiB3, denoted WhiB3TR, without the C-terminal residues (aa 91–102) containing the putative DNA binding motif to improve the protein stability and crystallizability. Phasing was performed using single-wavelength anomalous diffraction (SAD) of the iron–sulfur cluster containing four iron and four sulfur ions [4Fe-4S] cluster in the WhiB3:σ^A^_4_-β_tip_ complex (see Experimental procedures, Table 1 and Fig. S2). Two crystal forms were observed from the same crystallization drop, with the larger crystals in the P4_3_2_1_2 form and the small ones in the R3 form (Fig. 1*A*). The final model of WhiB3:σ^A^_4_-β_tip_ was refined to 1.35 Å in the P4_3_2_1_2 form and 1.5 Å in the R3 form.

**Table 1:** Data collection and refinement statistics

Proteins	WhiB3TR:σ^A^_4_-β_tip_ P4_3_2_1_2 (phasing)	WhiB3TR:σ^A^_4_-β_tip_ P4_3_2_1_2	WhiB3TR:σ^A^_4_-β_tip_ R3	WhiB3FL:σ^A^_4_-β_tip_:*P*_*whiB7*_
Data collection^a^				
Space group	P4_3_2_1_2	P4_3_2_1_2	R3	P2_1_2_1_2_1_
Cell dimensions				
*a*, *b*, *c* (Å)	122.2, 122.2, 115.1	121.8, 121.8, 114.3	120.8, 120.8, 31.8	50.7, 72.9, 95.4
α, β, γ (°)	90.0, 90.0, 90.0	90.0, 90.0, 90.0	90.0, 90.0, 120.0	90.0, 90.0, 90.0
Wavelength (Å)	1.7220	0.9795	0.9795	0.9787
Resolution (Å)	50–1.96 (1.99–1.96)	50–1.35 (1.37–1.35)	50–1.50 (1.53–1.50)	50–2.45 (2.49–2.45)
*R*_merge_	0.099 (1.094)	0.082 (3.02)	0.077 (2.16)	0.128 (1.344)
*I*/σ*Ι*	48.1 (3.7)	48.2 (1.2)	45.4 (1.6)	25.4 (1.2)
Completeness (%)	100.0 (100.0)	99.9 (100.0)	99.8 (99.7)	99.4 (94.6)
Multiplicity^b^	49.8 (48.8)	25.3 (24.7)	18.1 (14.2)	15.3 (6.9)
No. unique reflections	62,861 (3092)	187,184 (18,429)	27,420 (2696)	13,547 (617)
CC_1/2_(%)	99.1 (95.3)	99.9 (65.9)	100.0 (74.1)	98.7 (71.3)
Refinement				
Resolution (Å)		50–1.35 (1.40–1.35)	50–1.50 (1.56–1.50)	50–2.45 (2.53–2.45)
No. molecules per asymmetric unit		3	1	1
*R*_work_/*R*_free_		0.152/0.174	0.183/0.212	0.211/0.243
Included residue No.				
WhiB3		15–89; 15–89; 14–89^c^	6–90	16–90
σ^A^_4_		446–528	446–528	450–525
β_tip_		815–826	815–827	815–826
His-tag		1–6	1–6	-
No. atoms		5398	1689	1974
Macromolecules		4696	1555	1958
Ligand		180	29	13
Water		522	105	3
*B*-factors (Å^2^)		30.1	41.3	80.7
Macromolecules		28.4	41.2	76.6
Ligand		38.3	38.8	79.3
Water		42.5	43.9	66.2
r.m.s deviations				
Bond lengths (Å)		0.012	0.01	0.018
Bond angles (°)		1.68	1.29	2.29
Ramachandran statistics				
Favored regions (%)		98.14	97.89	91.77
Allowed regions (%)		1.30	1.58	8.23
Outliers (%)		0.56	0.53	0
PDB code		8CWT	8CWR	8CYF

a The highest resolution shell statistics are shown in parentheses.

b For the data used for SAD phasing, the anomalous multiplicity was shown.

c The three sets of the residues are for each of the three complexes in the asymmetric unit.

Like the other two σ^A^_4_-bound Wbl proteins, the WhiB3:σ^A^_4_-β_tip_ complex exists as a single complex in solution determined by size-exclusion chromatography (25, 26). However, in the crystals, the His_6_-tags in the N-terminal σ^A^_4_-β_tip_ of three WhiB3:σ^A^_4_-β_tip_ complexes are “glued” together by multiple nickel ions from the crystallization solution, resulting in a trimer of the WhiB3:σ^A^_4_-β_tip_ complexes in both crystal forms (Figs. 1*B* and S2*A*). Three WhiB3:σ^A^_4_-β_tip_ complexes in the trimer form are found in the asymmetric unit of the P4_3_2_1_2 structure. The R3 structure contains one WhiB3:σ^A^_4-_β_tip_ complex per asymmetric unit, as the trimer axis coincides with the crystallographic threefold axis. Correlated with the trimerization in the crystal form, we observed two structural re-arrangement in the WhiB3-bound σ^A^_4_-β_tip_ compared to that bound to WhiB7. First, the N-terminal residues (aa 446–456) of σ^A^_4_ in the WhiB3:σ^A^_4_-β_tip_ trimer form a β-hairpin with the His_6_-tag residues, instead of being part of helix h_s_1 of σ^A^_4_ as expected (Figs. 1*B* and S2*B*) (26, 31). Because the residues 446 to 456 of σ^A^_4_ are far from the WhiB3 binding site, we do not anticipate this structural change affects the mode of WhiB3 binding to σ^A^_4_. Second, β_tip_ in the WhiB3:σ^A^_4_-β_tip_ complex, which was expected to form intramolecular contacts with σ^A^_4_ in the σ^A^_4_-β_tip_ chimera, sticks into a neighboring protomer of the trimer and forms intermolecular interactions with σ^A^_4_ in a second WhiB3:σ^A^_4_-β_tip_ complex (Figs. 1*C* and S2*B*). The resulting σ^A^_4_-β_tip_ʹ, however, resembles σ^A^_4_-β_tip_ observed in the WhiB7:σ^A^_4_-β_tip_ complex and the RNAP holoenzyme (Fig. S3*C*) (26, 31), and thus it is used for the following structural analysis.

The crystal structures of the WhiB3:σ^A^_4_-β_tip_ complex in the P4_3_2_1_2 and R3 forms are essentially identical except for the N-terminal loop region, with an average Cα root-mean-square deviation of 0.27 Å for WhiB3 and 0.42 Å for σ^A^_4_, respectively (Fig. S3, *A* and *B*). The R3 structure was used for the following structural analysis of the WhiB3:σ^A^_4_-β_tip_ complex because of the better-defined electron density of the N-terminal residues (aa 6–13) in WhiB3 (Fig. S3, *D* and *E*).

### Molecular interface of the WhiB3:σ^A^_4_-β_tip_ complex compared to other σ^A^_4_-bound Wbl proteins

The overall architecture of the WhiB3:σ^A^_4_-β_tip_ complex is comparable to the previously reported WhiB1:σ^A^_4_ and WhiB7:σ^A^_4_-β_tip_ complexes. In all three cases, the molecular interface between the Wbl protein and σ^A^_4_ is hinged at the [4Fe-4S] cluster (Fig. 1*C*) (25, 26). The 4Fe-4S cluster binding pocket in WhiB3:σ^A^_4_-β_tip_ is enclosed (Fig. 1*D*), similar to that of WhiB1:σ^A^_4_ and in contrast to the solvent-accessible cluster in the case of WhiB7:σ^A^_4_-β_tip_.

**Figure 2:**
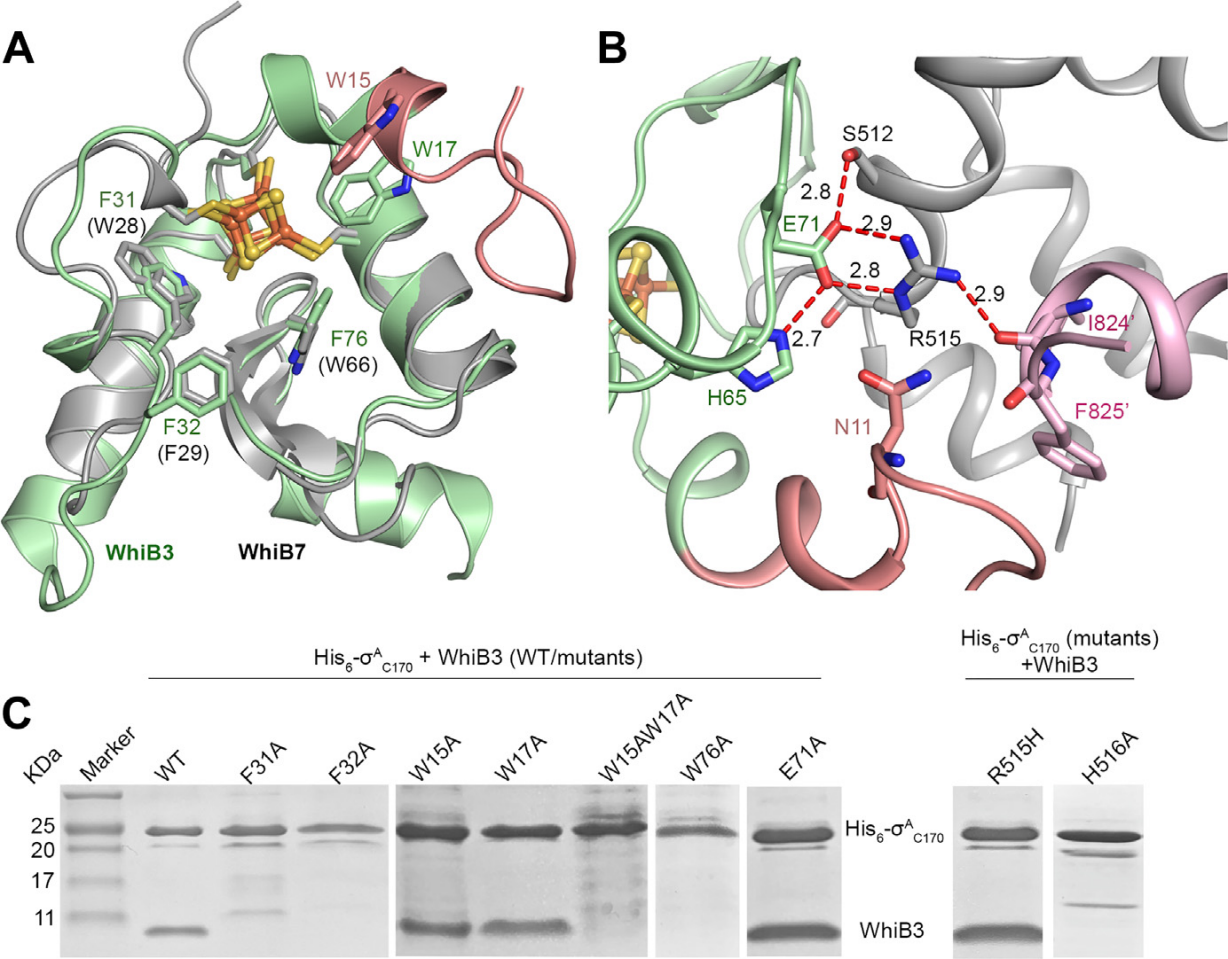
**Molecular interface between WhiB3 σ**^**A**^_**4**_**.***A*, a structural overlay of σ^A^_4_-β_tip_^ʹ^-bound WhiB3 (*pale green*, with the N-terminal region in *salmon pink*) and WhiB7 (PDB ID: 7KUG, *gray*). Only Wbl proteins are shown for clarity. *B*, close-up view of the hydrophilic network at the molecular interface of WhiB3 (*pale green*) and σ^A^_4_ (*gray*). The β_tip_^ʹ^ residues interacting with σ^A^_4_ in this region are shown in *pink*. *C*, SDS-PAGE analyses of the samples from the co-expression and affinity purification of tagless WhiB3 and His_6_-σ^A^_C170_ (either wildtype [WT] or mutant as indicated). σ^A^_4_-β_tip_, σ^A^_4_ fused with β_tip_ by an artificial linker.

Complex formation between WhiB3 and σ^A^_4_ is driven by the conserved aromatic residues near the [4Fe-4S] cluster binding pocket (Figs. 2*A* and S1*B*), as previously observed in the cases of WhiB1 and WhiB7 (25, 26). A single Ala substitution of F31, F32, or W76 in WhiB3 (corresponding to F17, F18, and W60 in WhiB1, respectively) or H516 in σ^A^_4_ completely abolishes the complex formation in the pull-down assays (Fig. 2*C*). To our initial surprise, a W17A mutation in WhiB3 does not abolish σ^A^_4_ binding in the pull-down assay (Figs. 2*C* and S4). W17 is invariant in the WhiB3 subclass, corresponding to the invariant W3 in WhiB1. It has been shown to play a crucial role in Fe–S cluster stability and complex formation in WhiB1:σ^A^_4_, while the absence of a W3 counterpart in WhiB7 leads to a solvent-accessible Fe–S cluster with increased O_2_ sensitivity in the WhiB7:σ^A^_4_ complex (25, 26). Subsequent sequence analysis reveals an additional conserved Trp in the WhiB3 subclass, W15, which is close to W17 and the Fe–S cluster binding pocket (Figs. 2*A* and S1*B*) and thus may compensate for the loss of W17 in the WhiB3 W17A mutant based on our pull-down assay. As shown in Figure. 2*C* and Fig. S4, although a single W15A mutation does not affect σ^A^_4_ binding, the double mutation of W15A and W17 A in WhiB3 completely abolishes the interaction in the pull-down assays. The existence of redundant Trp residues in the N-terminal WhiB3 highlights their importance in the complex formation, consistent with the observations from our studies on WhiB1 and WhiB7 (25, 26).

Both the WhiB3 and WhiB7 subclasses contain a similar triplet motif (“**E**P**Y**” in WhiB3 and “**EPW**” in WhiB7, respectively) immediately upstream of the β-turn, in which Glu forms hydrophilic interactions with σ^A^_4_ (Fig. S1*B*) (26). However, these motifs contribute differently to the complex formation. E61 in the “EPW” motif of WhiB7 orchestrates a hydrogen bond network at the molecular interface of the WhiB7:σ^A^_4_ complex and is required for σ^A^_4_ binding *in vitro* and the WhiB7-dependent antibiotic resistance in mycobacteria (21, 26). In contrast, E71 in the “EPY” motif of WhiB3 is part of the multicentered polar interaction networks involving N11, H65, and E71 in WhiB3, S512, and R515 in σ^A^_4_, as well as the backbone O of I824 and F825 in β_tip_ (Fig. 2*B*). Accordingly, either a WhiB3-E71A or σ^A^-R515H mutant does not affect the complex formation of WhiB3:σ^A^_4_ in the pull-down assays (Fig. 2*C*). Notably, the σ^A^_4_-R515H mutant was previously shown to affect the interaction between σ^A^_4_ and WhiB3 in yeast two-hybrid assay (18). The discrepancy observed here might be due to the different sensitivity between the two techniques. Nonetheless, the results from our pull-down assays indicate that the contribution of the polar interactions between WhiB3 E71 and σ^A^ R515 to the formation of the WhiB3:σ^A^_4_ complex is not as significant as the hydrophobic interactions between the conserved aromatic residues as described above.

**Figure 3:**
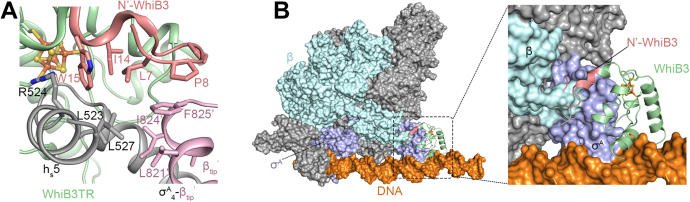
**Interactions between N-terminal WhiB3 and σ**^**A**^_**4**_**-β**_**tip**_**.***A*, a hydrophobic interface among N-terminal residues (aa 6–11) of WhiB3 (*pale green* with N-terminal residues in *salmon pink*), σ^A^_4_ (*gray*), and β_tip_ (*pink*). β_tip_ from the neighboring complex molecule (β_tip_^ʹ^) is shown to reflect its interaction with σ^A^_4_ and WhiB3 in the WhiB3TR:σ^A^_4-_β_tip_ complex. The hydrophobic residues at the molecular interface are shown in sticks. *B*, an overlay of σ^A^_4_ in the WhiB3:σ^A^_4_-β_tip_ complex with the WhiB7-RNAP-DNA complex in the closed state (PDB ID: 7KIM, σ^A^ colored *purple blue*, the α-subunits in *gray*, the β-subunit in *cyan*, and DNA in *orange*). The WhiB3:σ^A^_4_-β_tip_ complex is shown in the cartoon representations, and the WhiB7–RNAP–DNA complex is shown in the surface representation. Only WhiB3 (*pale green* with the N-terminal WhiB3 in *salmon red*) in the WhiB3:σ^A^_4_-β_tip_ complex is shown for clarity. By comparison, the N-terminal WhiB7 points toward an opposite direction relative to the N terminus of WhiB3 and interacts with the β^ʹ^-subunit of RNAP (Fig. S5). σ^A^_4_-β_tip_, σ^A^_4_ fused with β_tip_ by an artificial linker; RNAP, RNA polymerase; WhiB3TR, a truncated WhiB3 without C-terminal loop region (aa 91–102).

The N-terminal residues of WhiB3 before the first Cys in the Fe–S cluster binding motif are more conserved relative to WhiB7, while WhiB1 has an unusually short N terminus (Fig. S1) (25, 26). In the R3 crystal structure, the N-terminal residues 6 to 16 of WhiB3 interact with both σ^A^_4_ and β_tip_ outside the Fe–S cluster binding pocket (Fig. 3). The buried surface area between WhiB3 and σ^A^_4_-β_tip_ (1075.3 Å^2^) is significantly larger than that of the WhiB1:σ^A^_4_ complex (645 ± 27 Å^2^) and WhiB7:σ^A^_4_-β_tip_ complex (576 ± 3 Å^2^) (see Experimental procedures) (26). The net increase in the surface contact between WhiB3 and σ^A^_4_-β_tip_ is mainly attributed to the N-terminal residues 6 to 16 of WhiB3, which has a buried surface area of 372.1 Å^2^ with σ^A^_4_-β_tip_. In particular, L7, P8, I14, and W15 of WhiB3 form a hydrophobic core with both σ^A^_4_ and β_tip_ (Figs. 3*A* and S1*B*). When superposing the WhiB3:σ^A^_4_-β_tip_ structure onto the cryo-EM structure of the WhiB7–RNAP–DNA complex in the closed state (W-RPc, PDB ID: 7KIM) (27), the N-terminal WhiB3 is expected to interact with both σ^A^ and the β-subunit of RNAP (Fig. 3*B*). Interestingly, the N terminus of WhiB3 points to an opposite direction relative to that of the N-terminal WhiB7, which also extends into the RNA polymerase but interacts with the β^ʹ^-subunit of RNAP in the WhiB7-RNAP-DNA complex (Fig. S5) (27). The observation that the conserved N-terminal WhiB3 forms both hydrophobic and hydrophilic interactions with σ^A^_4_ and β_tip_ in the structural analysis (Figs. 2*B* and 3A) warrants further investigation of its effects on the WhiB3 binding to RNAP and transcriptional regulation.

### DNA binding by the WhiB3:σ^A^_4_-β_tip_ complex

**Figure 4:**
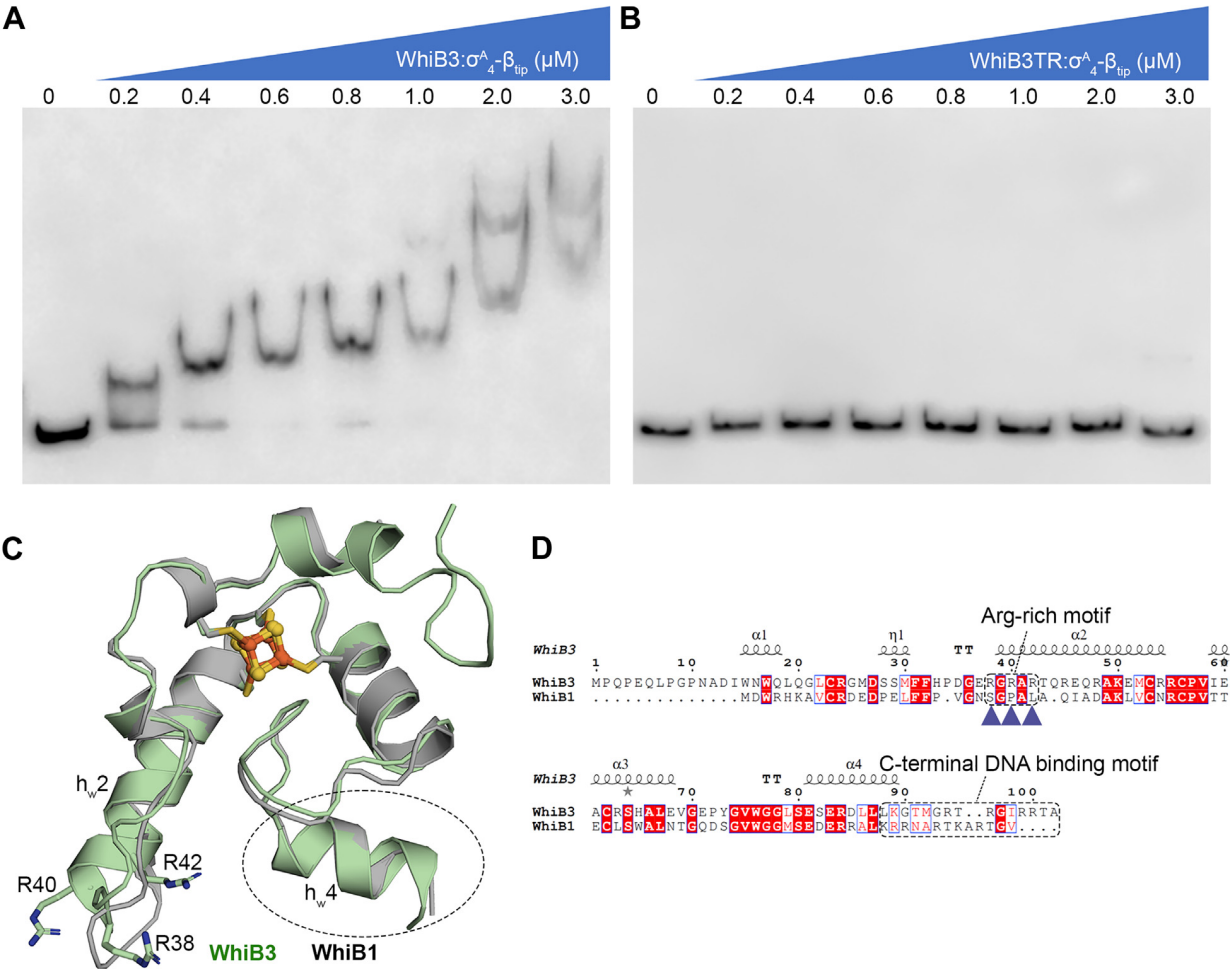
**Effect of the C-terminal WhiB3 on DNA binding.***A* and *B*, the EMSAs of the WhiB3:σ^A^_4-_β_-tip_ complex with the *pks3* promoter, either with wildtype WhiB3 protein (*A*) or the truncated WhiB3 without the C-terminal restudies 91 to 102 (WhiB3TR, *B*). *C*, an overlay of WhiB3 (*pale green*) in the WhiB3:σ^A^_4_-β_tip_ with the σ^A^_4_-bound WhiB1 (PDB ID: 6ONO, *gray*). Only Wbl proteins are shown for clarity. *D*, sequence alignment between *Mtb* WhiB3 and WhiB1. The putative C-terminal DNA binding motifs in the Wbl proteins and the conserved Arg-rich motif specific to the WhiB3 subclass are highlighted by *dashed rectangles*. The two invariant Arg residues (R38 and R42) in the conserved Arg-rich motif of WhiB3 and absent in WhiB1, are indicated by *blue triangles* (Fig. S1). R40 is a variant in the WhiB3 subclass. σ^A^_4_-β_tip_, σ^A^_4_ fused with β_tip_ by an artificial linker; EMSA, electrophoretic mobility shift assay.

A prior study by Singh *et al.* (12) has shown that WhiB3 binds to the promoter of the *pks2* and *pks3* genes to regulate the biosynthesis of major complex polyketides in *Mtb*. In this study, the oxidized but not the reduced WhiB3 in the cluster-free (apo-) form was found to bind DNA with high affinity but low specificity, and the interaction is sensitive to high concentrations of NaCl in the electrophoretic mobility shift assays (EMSAs). Holo-WhiB3 was also bound to DNA in the EMSAs but with lower affinity (with an observed shift at ∼0.8 μM), resulting in only a marginal DNA mobility shift. Since the previous studies on WhiB7 have shown that the DNA binding motifs in both WhiB7 and σ^A^_4_ coordinate DNA binding (26, 27), we thus tested whether that is also the case for WhiB3 by the EMSA using the *pks3* promoter as previously reported (12). As shown in Figure 4*A*, the WhiB3:σ^A^_4_-β_tip_ complex binds to the *pks3* promoter at concentrations as low as 0.2 μM in the EMSA, of which the binding affinity is higher than previously reported for holo-WhiB3 alone and thus implies a cooperative DNA binding by WhiB3 and σ^A^_4_ similarly to WhiB7 (12). The multiple DNA shifts observed in the EMSAs with a 316-bp *P*_*pks3*_ DNA, but not with a shorter DNA (22 bp), indicate that WhiB3:σ^A^_4_-β_tip_ binds to different sites on the long *P*_*pks3*_ DNA (Figs. 4*A* and S6*A*), reconciling with the low DNA specificity of WhiB3. We also find by the EMSAs that the C-terminal WhiB3 (aa 91–102) is important for DNA binding, as deletion of these residues abolishes DNA binding under the experimental conditions (Fig. 4*B*).

The observed DNA binding activity of σ^A^_4_-bound WhiB3 is in stark contrast with WhiB1. Thus far, only apo-WhiB1 has been shown to bind to its own promoter and several other target genes, and the basic residues (such as R74) in the C-terminal loop are required for DNA binding (22, 25, 32, 33). Neither holo-WhiB1 nor the WhiB1:σ^A^_4_ complex has shown DNA binding activity despite the extensive efforts. Our initial structural analysis did not uncover the structural basis that may account for the differences in DNA binding between σ^A^_4_-bound WhiB3 and WhiB1. The σ^A^_4_-bound WhiB3 and WhiB1 are strikingly similar, with an average Cα root-mean-square deviation of 0.54 in the 3D structural overlay (Fig. 4*C*). In particular, the structural arrangement of helix h_w_4 immediately adjacent to the putative C-terminal DNA-binding motif is essentially identical in the two structures, and an equal number of basic residues (four Arg and one Lys) is found in the C-terminal loop of WhiB1 and WhiB3 (Fig. 4, *C* and *D*). However, a closer examination of the WhiB3 subclass-specific sequences reveals an Arg-rich motif (corresponding to 38-**R**GRA**R**-42 in *Mtb* WhiB3) near the N-terminal helix h_w_2 that points toward the interface with DNA (Figs. 4, *C* and *D* and S1*B*). The role of this motif for WhiB3 binding to DNA has not yet been examined.

### Characterization of the conserved Arg-rich DNA-binding motif in WhiB3

**Figure 5:**
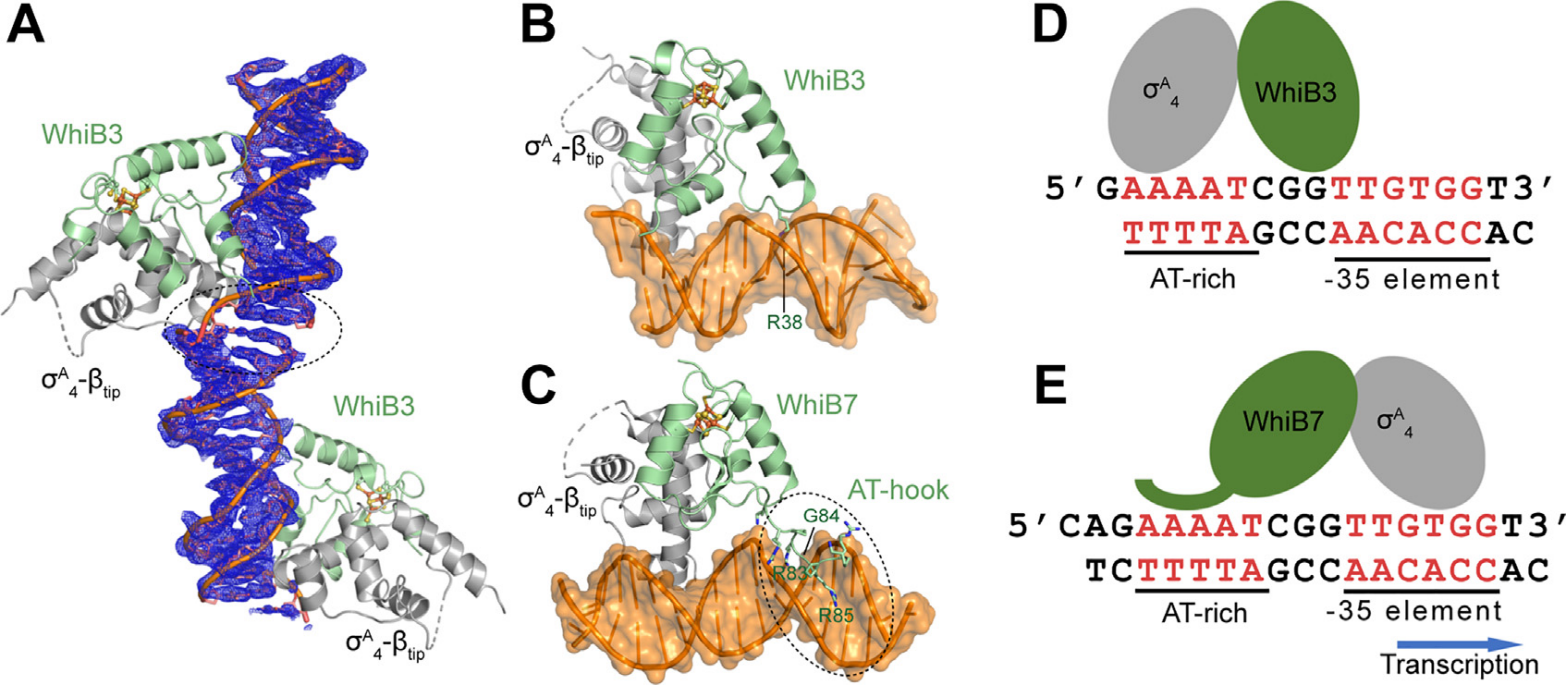
**Comparison of the *P***_***whiB7***_**binding site between WhiB3:σ**^**A**^_**4-**_**β**_**-tip**_**and WhiB7:σ**^**A**^_**4-**_**β**_**-tip**_**.***A*, simulated-annealing composite omit map around the *whiB7* promoter (*P*_*whiB7*_) in the crystal structure of two adjacent WhiB3:σ^A^_4_-β_tip_:*P*_*whiB7*_ complex molecules, contoured at 1.0 σ. The gap in the electron density map between the two adjacent *P*_*whiB7*_ DNA molecules is highlighted by a *black dash circle*, indicative of the correct assignment of the *P*_*whiB7*_ DNA. *B* and *C*, a side-by-side comparison of the crystal structures of WhiB3:σ^A^_4_-β_tip_:*P*_*whiB7*_ and WhiB7:σ^A^_4_-β_tip_:*P*_*whiB7*_ (PDB ID: 7KUF), respectively. The Wbl residues (R38 of WhiB3; R83-G84-R85 of WhiB7) inserted into the minor groove of the DNA helix are labeled. *D* and *E*, cartoon illustrations of the different modes of *P*_*whiB7*_ binding by WhiB3:σ^A^_4_-β_tip_ and WhiB7:σ^A^_4_-β_tip_, respectively. The *blue arrow* indicate the direction of the transcription in WhiB7:σ^A^_4_-β_tip_:*P*_*whiB7*_. In all the structures, WhiB3 and WhiB7 are colored *pale green*, σ^A^_4_-β_tip_ in *gray*, and the *P*_*whiB7*_ DNA in *orange*. σ^A^_4_-β_tip_, σ^A^_4_ fused with β_tip_ by an artificial linker.

To reveal the structural basis of the WhiB3:σ^A^_4_-β_tip_ complex binding to DNA, we attempted cocrystallization of WhiB3:σ^A^_4_-β_tip_ with the promoter DNA of either *pks3*, *apt,* or *whiB6*, all of which have been previously suggested to be under the regulation of WhiB3 (10, 12, 16, 34). Unfortunately, none of the hits from the crystallization screen led to high-resolution diffraction data for confident structure determination. Since WhiB3 possesses a putative AT-hook-like motif, out of curiosity, we tested and confirmed that the WhiB3:σ^A^_4_-β_tip_ complex binds to the *whiB7* promoter DNA (*P*_*whiB7*_) (Fig. S6*B*). We then cocrystallized the WhiB3:σ^A^_4_-β_tip_ complex with *P*_*whiB7*_ that was used in the crystallographic study of the WhiB7:σ^A^_4_-β_tip_:*P*_*whiB7*_ complex (26), which enabled us to collect high-quality diffraction data from a WhiB3:σ^A^_4_-β_tip_:*P*_*whiB7*_ crystal and refined the crystal structure at 2.45-Å resolution with the final R_free_/R_work_ value of 0.211/0.243 (see Experimental procedures). The well-defined electron density map allows us to unambiguously assign the nucleotides in the DNA helix of the complex crystal structure (Fig. 5*A*).

A structural comparison shows that although both WhiB3 and σ^A^_4_ in the WhiB3:σ^A^_4_-β_tip_:*P*_*whiB7*_ structure are involved in DNA binding like the case of WhiB7, they are dramatically different in how the Wbl-bound σ^A^_4_ interacts with *P*_*whiB7*_ (Fig. S5, *B*–*E*) (26, 27). σ^A^_4_ of the WhiB3:σ^A^_4_ complex orients about 180° relative to the WhiB7-bound σ^A^_4_ along with the *P*_*whiB7*_ DNA and inserts into the major groove upstream of the AT-rich WhiB7 binding site instead of the expected −35 element. The σ^A^_4_ binding site to the −35 element of *P*_*whiB7*_ has been previously confirmed in both the crystallographic and cryo-EM studies (26, 27). Therefore, the observation that σ^A^_4_ in the WhiB3:σ^A^_4_-β_tip_:*P*_*whiB7*_ complex binds to a physiologically irrelevant site of *P*_*whiB7*_ opposite to the direction of transcription initiation indicates that *P*_*whiB7*_ is unlikely a target of WhiB3. However, the crystal structure of the WhiB3:σ^A^_4_-β_tip_:*P*_*whiB7*_ complex provides valuable insights into the general features of how WhiB3 interacts with DNA, which is the primary focus of our structural analysis below.

**Figure 6:**
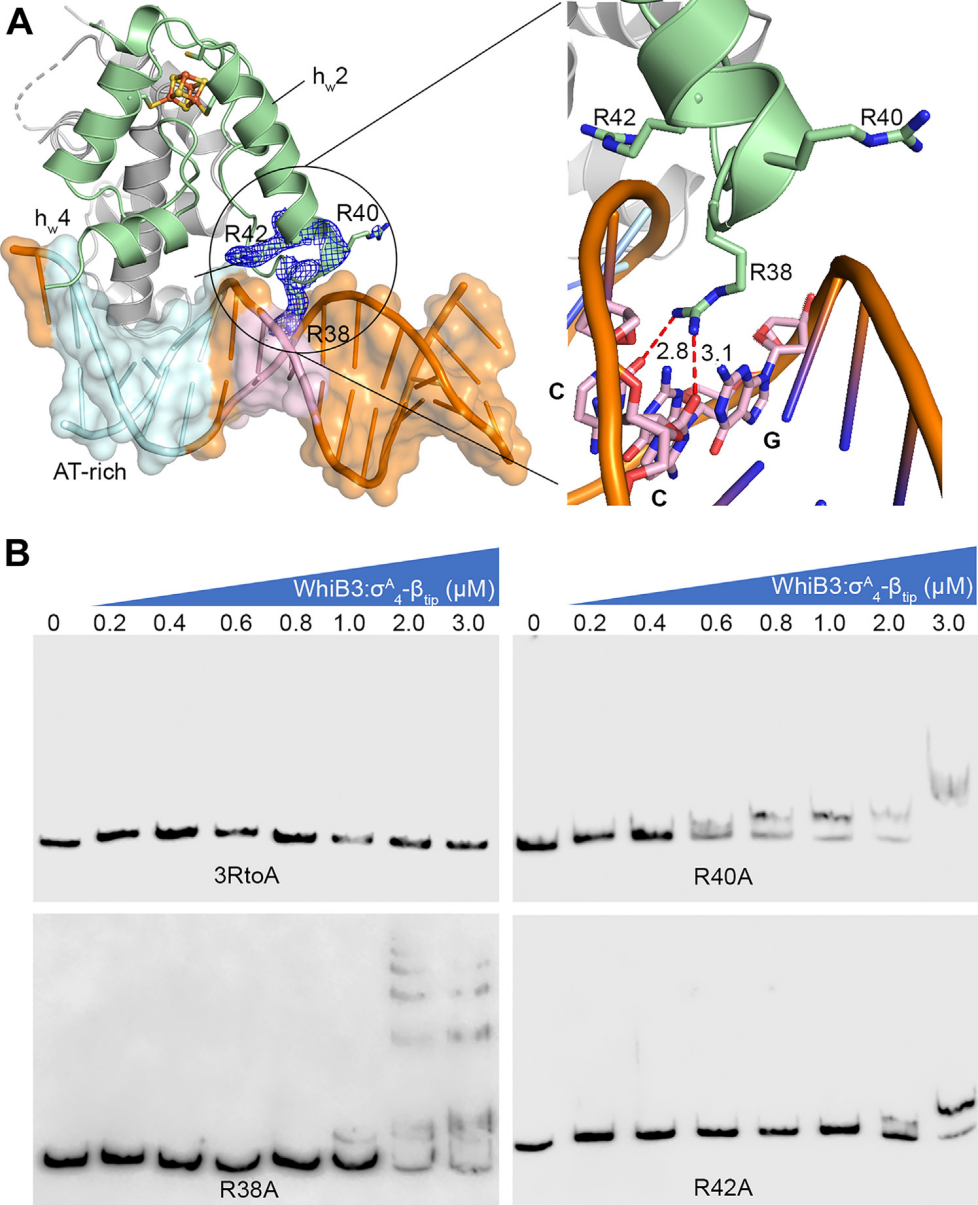
**Identification of a conserved Arg-rich DNA binding motif in WhiB3.***A*, highlights of the hydrophilic interactions between the conserved Arg-rich motif of WhiB3 and DNA in the WhiB3:σ^A^_4_-β_tip_:*P*_*whiB7*_ complex. The AT-rich DNA sequence in *P*_*whiB7*_ is colored cyan, with the rest of the DNA in *orange*, WhiB3 in *pale green*, and σ^A^_4_-β_tip_ in *gray*. The three Arg residues (*i.e*., R38, R40 and R42) in the conserved Arg-rich motif of *Mtb* WhiB3 are shown in *stick representations*, with the 2Fo-Fc density map contoured at 1.0 σ. *B*, EMSAs of the σ^A^_4_-β_tip_-bound WhiB3 (wildtype or mutant as indicated) with the *pks3* promoter DNA. All the three Arg residues are substituted by Ala in the WhiB3-3RtoA mutant. σ^A^_4_-β_tip_, σ^A^_4_ fused with β_tip_ by an artificial linker; EMSA, electrophoretic mobility shift assay.

Our structural and biochemical analyses show that WhiB3 engages with DNA differently from WhiB7, and the newly identified Arg-rich motif in WhiB3 as described above plays a central role in DNA binding (Table S2; Figs. 5 and S7) (26, 27). For the case of WhiB7, the central RGR motif of the WhiB7 AT-hook lies in the minor groove of the A-track sequence upstream of the −35 element and forms both hydrophilic and hydrophobic interactions with the A/T nucleotides as well as the sugar and phosphate groups of the DNA. Although a “RGR” sequence is also present in the Arg-rich motif (“**R**GRA**R”**) of *Mtb* WhiB3, the RGR sequence is not conserved in the WhiB3 subclass and only R38 in the motif inserts vertically into the minor groove of the junction between the WhiB7 binding site and the −35 element. R38 forms polar contacts with the nucleotides of 2 GC pairs (Table S2; Figs. 6, *A* and *B*; Fig. S7), in contrast to the preference of AT-rich DNA by the WhiB7 AT-hook. The other conserved residue in the Arg-rich motif, R42, is near the edge of the DNA duplex (∼4 Å away) and thus may form hydrophilic interactions with the phosphate backbone in solution. The variant residue R40 is also near the DNA helix. However, it is unclear how it interacts with DNA because of the poor electron density of the side chain, implying a nonspecific interaction with the *P*_*whiB7*_ DNA and echoing the low conservation of this residue in the WhiB3 subclass. We notice reduced DNA contacts for the residues in the WhiB3-bound σ^A^_4_ (*e.g.*, R478 and T488) compared to that bound to WhiB7, coincident with the observation that WhiB3-bound σ^A^_4_ being displaced off the −35 binding site of *P*_*whiB7*_. The differences observed in how σ^A^_4_ -bound WhiB3 and WhiB7 bind to *P*_*whiB7*_ are in agreement with our analysis of the *P*_*whiB7*_ DNA structure between the two complexes. As shown in Fig. S8, the central minor-groove width (∼3.5 Å) around the AT-rich region of *P*_*whiB7*_ in WhiB3:σ^A^_4_-β_tip_:*P*_*whiB7*_ is characteristic of A-track DNAs (35, 36) and is significantly narrower than that of WhiB7:σ^A^_4_-β_tip_:*P*_*whiB7*_ (∼7 Å) where WhiB7-bound to the minor groove of the AT-rich region. Additionally, the break of base–base stacking between the −36 and −37 nucleotide, indicating DNA bending as a result of the cooperative action of WhiB7 binding to the AT-rich region and σ^A^_4_ binding to the adjacent −35 hexamer (26), is absent from *P*_*whiB7*_ in the WhiB3:σ^A^_4_-β_tip_:*P*_*whiB7*_ complex (Fig. S7). However, it should be noted that since σ^A^_4_ binds to the major groove immediately upstream of the AT-rich region, and *P*_*whiB7*_ in the WhiB3:σ^A^_4_-β_tip_:*P*_*whiB7*_ is 2-bp shorter than in WhiB7:σ^A^_4_-β_tip_:*P*_*whiB7*_ near the AT-rich region, we cannot unequivocally attribute the cause of the observed differences.

Results from the EMSAs confirm our structural analysis that all three Arg residues in the conserved Arg-rich motif play a role in WhiB3 binding to the *pks3* promoter, while the difference in their contributions is noted (Fig. 6*B*). A triple Arg-to-Ala mutation of the Arg-rich motif (3RtoA) completely abolishes DNA binding in the EMSAs. Substitution of each Arg by an Ala significantly affects WhiB3 binding to the *pks3* promoter, with the effect of the R40A mutation relatively weaker than the other two (*i.e.*, R38A and R42A) consistent with the structural and sequence analysis as mentioned above. The C-terminal WhiB3 residues (aa 91–102), which are required for DNA binding in the EMSA (Fig. 4*B*), are not visible in the electron density map. It is possible that these C-terminal residues interact with DNA through nonspecific polar contacts and thus result in the ill-defined electron density in the crystal structure.

**Figure 7:**
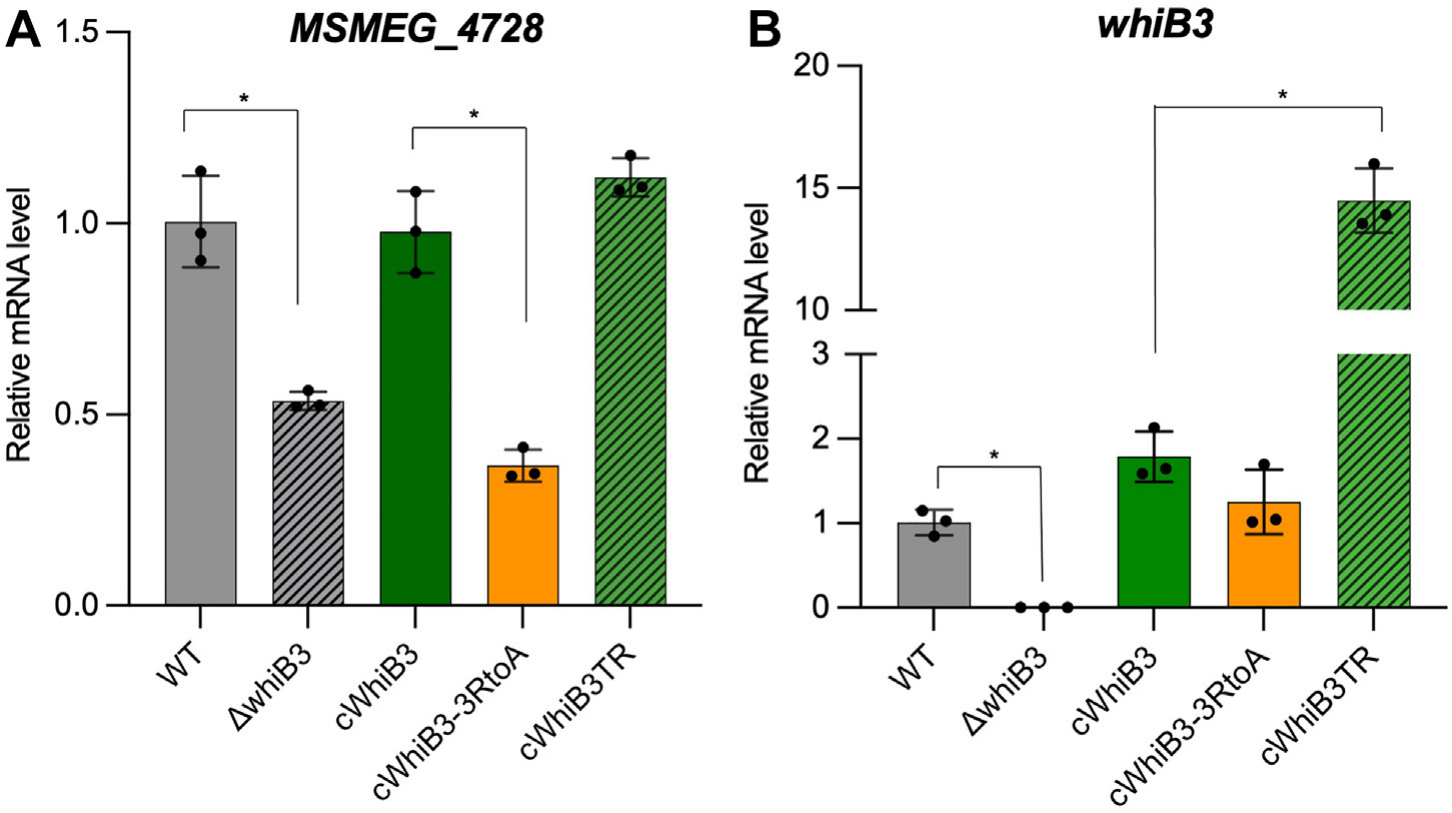
**Identification of the essential DNA binding motif for the WhiB3-dependent transcription activation in *Msm*.***A* and *B*, are the RT-qPCR analyses of relative mRNA levels of MSMEG_4728 and *whiB3*, respectively, in the *Msm* wildtype (wt), the *whiB*3 deletion mutant (*ΔwhiB3*) alone or complemented with the wildtype whiB3 (*cWhiB3*), the *whiB3-3RtoA* mutant (*cWhiB3-3RtoA*) or the C-terminal truncated *whiB3* (c*WhiB3TR*). The mRNA level in each sample was normalized to the level σ^A^, and the fold of changes were calculated relative to wt. Data are representative of three biological replicates. Statistical significance is determined by Student’s *t* test and displayed as ∗*p* < 0.05 in the comparisons as indicated. The error bars represent mean ± SD. *Msm, Mycobacterium smegmatis*; RT-qPCR, reverse transcription-quantitative polymerase chain reaction.

Consistent with the structural and biochemical analyses, our reverse transcription-quantitative polymerase chain reaction (RT-qPCR) study indicates that the conserved Arg-rich motif (“**R**GRA**R”**) is required for the WhiB3-dependent transcriptional regulation in *Mycobacterium smegmatis* (*Msm*) (see Experimental Procedures) (Fig. 7). As the previous study suggested (29), our results show that WhiB3 regulates the expression of MSMEG_4728 in *Msm*, which encodes a putative polyketide synthase-associated protein. The reduction of the mRNA levels of MSMEG_4728 in the *whiB3* deletion mutant (*ΔwhiB3*) can be complemented by the wildtype WhiB3 but not by the WhiB3-3RtoA mutant, indicating the essential role of the conserved Arg-rich motif (“**R**GRA**R”**) for WhiB3-dependent transcriptional activation (Fig. 7*A*). In contrast, deletion of the C-terminal WhiB3 (WhiB3TR) shows a negligible effect on the expression of MSMEG_4728 when compared to the wildtype. This observation indicates that the C-terminal region is not essential for the WhiB3-dependent transcriptional regulation, reconciling with the high variation of this region in the WhiB3 subclass and the nonspecific DNA binding in the complex structural analysis.

## Discussion

This study provides an atomic view of how WhiB3 interacts with σ^A^_4_ and DNA. Structural comparison of the WhiB3:σ^A^_4_ complex with the σ^A^_4_-bound WhiB1 and WhiB7 reveals that all the three Wbl proteins share a similar molecular interface with σ^A^_4_, and the subclass-specific structural features underlie the structural basis for DNA binding by WhiB3. Complemented by molecular and biochemical approaches, we uncover a conserved Arg-rich DNA binding motif near the N-terminal helix h_w_2 in the WhiB3 subclass and determine the importance of the C-terminal basic residues for DNA binding and transcription activation by *Mtb* WhiB3. It is important to note that some WhiB3 subclass members, such as those in *Streptomyces hygroscopicus* (WP_041665551), *Geodermatophilus obscurus* (WP_012950425), and *Beutenbergia cavernae* (WP_015883541), do not have any basic residues corresponding to the putative C-terminal DNA binding motif (aa 91–102) of *Mtb* WhiB3, underlying the significance of the newly identified Arg-rich motif for DNA binding by the WhiB3 subclass proteins. Furthermore, the identification of the conserved Arg-rich DNA-binding motif in WhiB3 provides a plausible explanation for the difference in DNA-binding activities between WhiB3:σ^A^_4_ and WhiB1:σ^A^_4_ and sheds light on the DNA binding preference of WhiB3.

It is well known that the −35 hexamer is much less conserved in mycobacterial promoters than in *E. coli* (37, 38, 39). Consistently, σ^A^_4_ does not bind to any DNA targets used in the assays without the DNA binding motif of a Wbl protein in the EMSAs (Fig. 6) (25, 26). In this context, the Wbl proteins, which exist exclusively in Actinobacteria but not in *E. coli*, may be employed by mycobacteria to serve as “guide dogs” to compensate for the low DNA affinity and specificity of σ^A^_4_ in the target-specific transcriptional regulation in response to environmental cues. Intriguingly, the mode of DNA binding by WhiB3 and WhiB7 are fundamentally different despite the similarity (*i.e.*, the “RGR” sequence) in their DNA binding motif. The central “RGR” motif of WhiB7 AT-hook lies in and grips the minor groove of the A-track sequence like a hand. The mode of WhiB7 AT-hook interaction with DNA provides the structural basis for how WhiB7 AT-hook binding opens the minor groove and reversely bends the A-track DNA to facilitate σ^A^_4_ binding to the −35 hexamer (26). Lacking a canonical AT-hook, WhiB3 R38 is the only DNA sequence discriminator and interacts with DNA *via* the guanidino group like finger touch, which explains the low DNA sequence specificity in the EMSAs and why WhiB3 cannot interact with the A-track sequence in the *P*_*whiB7*_ promoter like WhiB7. The observation that σ^A^_4_ is displaced from the consensus −35 element upon binding to WhiB3 hints that WhiB3 either strongly disfavors the A-track sequence or favors the GC nucleotides.

The results from the WhiB3:σ^A^_4_-β_tip_:*P*_*whiB7*_ structure analysis described above also provide the evidence accounting for the high salt sensitivity and low specificity of WhiB3 observed in this study and the previous report (12). These observations raise the question of how WhiB3 selectively regulates target-specific gene expression. Recent work on the ortholog of *Mtb* WhiB2 in *Streptomyces* (WhiB) and *Corynebacteria* (WhcD) indicates that it coordinates with another regulator WhiA for DNA binding and transcriptional regulation, while WhiB does not bind to DNA alone (40, 41, 42). Likely WhiB3 may also adopt this strategy for engaging with DNA and enhancing target specificity. Our speculation is in line with the observation that the C-terminal WhiB3, which is required for enhancing WhiB3 binding to DNA in the EMSAs, does not involve in specific DNA binding in our structural analysis and is dispensable for transcription activation in *Msm*. It is interesting to note that the absence of the C-terminal region disrupts WhiB3 binding to DNA in the EMSAs and leads to over 7-fold increase in the *whiB3* transcript level comparable to the wildtype WhiB3-complemented strain. Previous studies on WhiB1 have shown that the C-terminal Arg and Lys residues, which are also dispensable for holo-WhiB1-mediated transcriptional regulation, are required for DNA binding by apo-WhiB1 to repress several essential genes, including *whiB1* itself (25, 32, 33). Further study is needed to test whether apo-WhiB3 also utilize the C-terminal domain for DNA binding and self-repression.

It is noted that the core helices of Wbl proteins involved in σ^A^_4_ binding are highly conserved. However, the structural basis underlying the functional differences in the Wbl subclasses remains elusive. Our structural, biochemical, and molecular analyses of WhiB3 reported here provide crucial evidence for understanding how WhiB3 engages target DNAs differently from other characterized Wbl proteins (*i.e.*, WhiB1 and WhiB7) *via* the conserved Arg-rich motif in the middle loop and regulates target-specific gene expression in coordinate with σ^A^. A fuller view of these structural bases is critical for understanding the Wbl-dependent mechanism of pathogenesis and persistence in *Mtb* and building a stronger informational foundation for developing effective drugs for the treatment of the mounting threat of tuberculosis.

## Experimental procedures

### *E. coli* strains

All the *E. coli* strains were grown in Luria–Bertani media and at 37 °C, 200 rpm, unless otherwise specified.

### Mycobacterial strains

*Msm* MC^2^ 155 and the related mutant without and with a complementary gene generated in this study were grown in either Middlebrook 7H9 broth or on 7H10 agar (BD Difco) supplemented with 10% (v/v) ADS (2% dextrose, 5% bovine serum albumin and 0.85% NaCl), 0.2% (v/v) glycerol, 0.05% (v/v) Tween 80 (Sigma). When appropriate, the media were supplemented with antibiotics at following concentrations: hygromycin, 100 μg/ml for *E. coli* and 50 μg/ml for *Msm* and kanamycin, 50 μg/ml for *E. coli* and 25 μg/ml for *Msm*.

### Plasmid construction for protein overexpression in *E. coli*

The bacterial strains and plasmids used in this study are listed in Table S1. The genes encoding *Mtb* WhiB3 (Rv3416, 1–102 aa) and the C-terminal domain of σ^A^_4_ (Rv2703) containing the last 170 residues (aa 359–528, denoted σ^A^_C170_) were amplified from *Mtb* H37Rv genomic DNA (a gift from Dr Midori Kato-Maeda’s group at the University of California, San Francisco) by PCR, and subsequently cloned into pET21b(+) and pCDF-1b to express tagless WhiB3 and σ^A^_C170_ with a N-terminal His_6_-tag, respectively. The resulting plasmids, pET21-MtbWhiB3 and pCDF-1b-6HisMtbσ^A^_C170_, were subsequently modified for the overexpression of the desirable proteins with either truncation or point mutation used for crystallization and biochemical assays as described in the related sections. Briefly, the pET21b-MtbWhiB3TR plasmid was modified from pET21b-MtbWhiB3 by site-directed mutagenesis for the expression of a truncated WhiB3 (WhiB3TR, containing aa 1–90) without the last ten residues in the C terminus for crystallographic work and EMSA. As previously described (26), the pET28b-6HisMtbσ^A^_C82_-β_tip_ plasmid encoding the chimera protein His_6_-σ^A^_C82_-β_tip_ was used for crystallographic work, and the pET28b-6HisMtbσ^A^_C112_-β_tip_ plasmid was used for the expression of the chimera protein His_6_-σ^A^_C112_-β_tip_ in the EMSAs.

All the plasmids were confirmed by DNA sequencing before being transformed into *E. coli* BL21-Gold (DE3) strain for protein expression.

### General procedures for protein expression, purification, and analysis

Overexpression and purification of the proteins of interest from *E. coli* BL21-Gold (DE3) for structural and biochemical studies were done as described in our study of WhiB7 (26). Samples after each step of purification were analyzed by SDS-PAGE and by UV-Visible (UV-Vis) spectroscopy. Unless otherwise specified, the final purified proteins in 50 mM Tris-HCl, pH 8.0, 100 mM NaCl, and 0.2 mM tris(2-carboxyethyl)phosphine were stored in liquid nitrogen until use. UV-Vis spectra of the purified proteins were recorded using an HP 8452a diode array UV-Vis spectrophotometer (Agilent Technologies Inc). The absorption at 410 nm, characteristic of proteins containing [4Fe-4S]^2+^ clusters (43, 44), was used to estimate the occupancy of the Fe–S cluster in the protein samples containing a Wbl protein. Protein concentrations were estimated either by the Pierce Bradford Assay Kit (Thermo Fisher Scientific) or UV-Vis absorption spectroscopy.

### Pull-down assays

To verify the role of the residues in WhiB3 and σ^A^_4_ for the complex formation, the two plasmids encoding a tagless *Mtb* WhiB3 (wildtype or mutant) and a His_6_-σ^A^_C170_ (wildtype or mutant), respectively, were transformed into *E. coli* BL21-Gold (DE3) for protein coexpression and affinity purification by Ni-NTA Sepharose resin as previously described (26). The purified protein samples were analyzed by UV-Vis spectroscopy and SDS-PAGE.

### DNA-binding assays

Nondenaturing gel EMSAs were used to test the binding of *Mtb* WhiB3 (wildtype and mutants) in complex with His_6_-σ^A^_C112_-β_tip_ to the promoter of the *pks3* gene (*P*_*pks3*_) as previously described (25, 26). The *P*_*pks3*_ dsDNA containing 316 bp upstream of the start codon of the *pks3* gene, the same promoter region used in the previous EMSA (12), was amplified from *Mtb* H37Rv genomic DNA using the biotin-labeled primer pairs (forward primer: biotin-labeled 5′-AACGGATTTCGGGGCCTTTTGCGTCTGCT-3′; reverse primer: 5′-TTACCAACACATTCGGGCTCAGGAT-3′). In each of the 10-μl binding reactions, 0.2 nM biotin-labeled *P*_*pks3*_ dsDNA was incubated with σ^A^_C112_-β_tip_-bound *Mtb* WhiB3 (wildtype or mutant) in the presence of 25 mM Tris pH 8.0, 5 mM MgCl_2_, 0.1 mg/ml BSA, 1 mM dithiothreitol, and 20 mM KCl. After incubation at room temperature for 20 min, the samples were analyzed on 6% native polyacrylamide gels and UV crosslinked to Hybond-N+ nylon membrane (GE Healthcare Life Sciences) after gel transfer. The biotin-labeled DNA was detected using the LightShift Chemiluminescent EMSA Kit (Thermo Scientific, Inc) according to the manufacturer’s instructions.

### Creation of a *whiB3* deletion mutant in *Msm*

The *whiB3 (MSMEG_1597)* deletion mutant (*ΔwhiB3*) in *Msm* was generated using homologous recombination–based in-frame unmarked deletion as previously described (45). The resulting *ΔwhiB3* strain was verified by PCR and further confirmed by DNA sequencing.

### Creation of the *whiB3-complemented strains*

For the complementation test in the *ΔwhiB3* strain, the DNA fragment encoding *Msm*WhiB3 (wildtype or mutant) was cloned into the integration plasmid pKW08-Lx-Int (integrated plasmid) for expression of tagless *Mtb*WhiB3 from the native *whiB3* promoter (46). The resulting plasmids, pKW08-Int-PwhiB3-msmWhiB3, pKW08-Int-PwhiB3-msmWhiB3TR, and pKW08-Int-PwhiB3-msmWhiB3-3RtoA, are listed in Table S1. Each of these plasmids was transformed into the *ΔwhiB3* strain to determine whether it can complement WhiB3-dependent transcription by RT-qPCR.

### RT-qPCR

Msm cells were cultured in 50 ml 7H9 broth supplemented with 50 ug/ml hygromycin, if applicable, until *A*_600nm_ reached ∼0.7 to 0.8. The cells were then harvested, and RNA extraction was done using RNeasy Mini kit (QIAGEN) according to the manufacturer’s instructions. RNA was then treated with gDNA wipeout buffer supplied in the QuantiTect Reverse Transcription Kit (Qiagen). Complementary DNA was obtained by reverse transcription using the QuantiTect Reverse Transcription Kit from 1 μg of each of the RNA samples. Quantification PCR (qPCR) was performed using CFX Connect Real-Time PCR Detection System and SsoAdvanced Universal SYBR Green Supermix (Bio-Rad). All RT-qPCR reactions were performed in biological triplicates. The mRNA level of the *sigA* gene was used as the reference. The extent of expressional changes was calculated using the 2-ΔΔCt method and scaled to the Msm wildtype strain. The results were analyzed using the Origin software. The primers used for RT-qPCR were:

*sigA*: forward primer, 5′- GTGTGGGACGAGGAAGAGTC-3′

reverse primer, 5′- ACCTCTTCTTCGGCGTTGAG-3′

*whiB3:* forward primer, 5′-CAACTGCGACACATTTCCTTCGCAC-3′

reverse primer, 5′- GAATCCGAGCGTGAGCTTCTGC -3′

*MSMEG_4728*: forward primer, 5′- ACCGTTCCGGTGTGGAACAT-3′

reverse primer, 5′- CGGTGAACTCGAAACGGCTG-3′.

These primers were designed to be in the coding regions of the transcripts.

### Crystallization

Initial crystallization screens of the WhiB3TR:σ^A^_C82-_β_tip_ complex were carried out at 18 °C in a Coy anaerobic chamber using the sitting-drop vapor diffusion method, followed by optimization of the crystallization hits. High-quality crystals were obtained by mixing 1 μl WhiB3TR:σ^A^_4-_β_tip_ at 80 mg/ml with an equal volume of the reservoir solution containing 10 to 20 mM nickel chloride and 0.8 to 1.0 M lithium sulfate.

For the crystallization of the WhiB3:σ^A^_C82_-β_tip_:*P*_*whiB7*_ complex, 16-bp synthetic *P*_*whiB7*_ duplex DNA with a 5′ G/C overhang at each end (5′-GAAAATCGGTTGTGGT-3′/5′-TTTTAGCCAACACCAC-3′, Sigma-Aldrich) was used for cocrystallization with the WhiB3:σ^A^_C82_-β_tip_ complex. The WhiB3:σ^A^_C82_-β_tip_ complex was first mixed in a 1:1 M ratio with 100 μM P_*whiB7*_ and subsequently concentrated to 40 mg/ml before crystallization. The best crystals were obtained by mixing 1 μl of the protein–DNA complex with the reservoir solution containing 0.1 M calcium acetate, 0.1 M sodium cacodylate, pH 6.5, 10%∼13% polyethylene glycol 8000. All the crystals were briefly soaked in the reservoir solution supplemented with 20% glycerol for cryoprotection before flash-cooling in liquid nitrogen.

### X-ray crystallographic data collection, structural determination, and analysis

X-ray diffraction data were collected at the beamlines 9-2 and 12-2 of the Stanford Synchrotron Radiation Lightsource from single crystals maintained at 100 K using a 6M Pixel Array Detector. The diffraction data were collected at Se K-edge (12,658 eV) and used for the final structural refinement for all three structures. The SAD data collected from a single crystal of WhiB3TR:σ^A^_C82-_β_tip_ at the Fe K-edge absorption peak (7200 eV) were used for experimental phasing. The diffraction data were indexed, integrated, and scaled using HKL2000 (47). Model building and structure refinement were performed in COOT and Phenix (48, 49). The data collection and refinement statistics are summarized in Table 1.

Two forms of the WhiB3TR:σ^A^_C82-_β_tip_ crystals in space groups P4_3_2_1_2 and R3, respectively, were observed in the same crystallization drop. The phases for the P4_3_2_1_2 form were solved by SAD using Phenix.Autosol, with a figure of merit of 0.58. Model building and structure refinement were carried out using COOT and Phenix (48, 49). The final model in the P4_3_2_1_2 form was refined to 1.35 Å with one trimer of the WhiB3TR:σ^A^_C82-_β_tip_ complexes per asymmetric unit. The R3 crystal structure was solved by molecular replacement using a single copy of WhiB3TR:σ^A^_4-_β_tip_ from the P4_3_2_1_2 crystal structure as a search model and refined to 1.5 Å in the final model. The trimer axis in the R3 crystal structure coincides with the crystallographic threefold axis, resulting in one WhiB3:σ^A^_4-_β_tip_ complex per asymmetric unit. The WhiB3TR:σ^A^_4-_β_tip_ structures in the two crystal forms are essentially identical except for the N-terminal residues (aa 6–11) of WhiB3.

The phases for the WhiB3: σ^A^_4_-β_tip_:*P*_*whiB7*_ structure were determined by molecular replacement using the WhiB3:σ^A^_4_-β_tip_^ʹ^ structure in the R3 form as the search model. Phenix.autobuid was then used to build the first partial DNA model of *P*_*whiB7*_. The assignment of the rest nucleotides was done manually, followed by multiple cycles of refinements using COOT and PHENIX (48, 49).

## Data visualization

Sequence alignments were performed using Clustal Omega and ESpript online server (https://espript.ibcp.fr), and the sequence logo was generated using WebLogo (50, 51, 52). The representative WhiB3 subclass sequences used for the alignments are modified from the study by *Chandra et al.* (53) and listed in the legend of Fig. S1. 3D structure figures were prepared with the PyMol Molecular Graphics System v2.3 (https://pymol.org/2/). The molecular interface between WhiB3 and σ^A^_4-_β_tip_ʹ was estimated using the online macromolecular interface tool PISA (54).

## Data availability

Atomic coordinates and structure factors have been deposited in the RCSB Protein Data Bank (PDB) under the accession codes 8CWT and 8CWR for the WhiB3:σ^A^_4_-β_tip_ complex in the P4_3_2_1_2 form and the R3 form, respectively, and 8CYF for the WhiB3:σ^A^_4_-β_tip_:*P*_*whiB7*_ complex, respectively.

## Supporting information

This article contains supporting information (25, 26, 50, 54).

## Conflict of interest

The authors declare no conflict of interest with the contents of this article.
