# Supplementary material for: Structural basis of DNA binding by the WhiB-like transcription factor WhiB3 in Mycobacterium tuberculosis
Source: J Biol Chem. 2023 May 2;299(6):104777. doi: 10.1016/j.jbc.2023.104777 (PMC10245118; doi:10.1016/j.jbc.2023.104777)
Supplement: Supporting information [file mmc1.docx]

***Supplementary Materials for***

**Structural Basis of DNA Binding by the WhiB-Like Transcription Factor WhiB3 in *Mycobacterium tuberculosis***

Tao Wan^1^, Magdaléna Horová^1#^, Vimmy Khetrapal^1#^, Shanren Li^1,4^, Camden Jones^1^, Andrew Schacht^1,5^, Xinghui Sun^1^, LiMei Zhang^1,2,3*^

^1^Department of Biochemistry, University of Nebraska-Lincoln, Lincoln, NE, 68588, USA

^2^Redox Biology Center, University of Nebraska-Lincoln, Lincoln, NE, 68588, USA

^3^Nebraska Center for Integrated Biomolecular Communication, University of Nebraska-Lincoln, Lincoln, NE, 68588, USA

^4^Present address: College of Life Sciences, Fujian Normal University, Fuzhou, Fujian, 350117, China

^5^Present address: Division of Chemistry and Chemical Engineering, California Institute of Technology, Pasadena, CA, 91125, USA

^*^Correspondence:[lzhang30@unl.edu](mailto:lzhang30@unl.edu)

**Supplementary Table S1.** Bacterial strains and plasmids used in this study.

| **Label** | **Description** | **Reference** |
| --- | --- | --- |
| Bacterial Strains | | |
| *E. coli strains* | | |
| XL1-Blue | Host strain for routine cloning work | Stratagene |
| BL21-Gold (DE3) | Host strain for recombinant protein expression | Agilent Technologies |
| *Mycobacterium smegmatis (Msm)* | | |
| WT | *Mycobacterium smegmatis* MC2 155 (ATCC 700084), unmodified | ATCC |
| *∆whiB3* | An unmarked whiB3 deletion mutant in *Mycobacterium smegmatis* MC2 155 | This study |
| Plasmids | | |
| Plasmids for expression and purification of the target proteins from *E. coli* | | |
| pCDF-1b-His_6-_σ^A^_C170_ | The DNA fragment encoding the C-terminal domain of the *Mtb* *σ^A^* (last 359-528 aa) was amplified from *Mtb* H37Rv genomic DNA and inserted into the KpnI-XhoI site of pCDF-1b for expressing His_6_-σ^A^_C170_; Spectinomycin (Spec^+^) | (1) |
| pCDF-1b-His_6_-σ^A^_C170_ mutant (R515H, H516A) | A modification of pCDF-1b-His_6-_σ^A^_C170_ by site-directed mutagenesis for expressing His_6_-σ^A^_C170_ with a single mutation of either R515H or H516A; Spec^+^ | (1) |
| pET28b-6HisMtbσ^A^_C112_-β_tip_ | The gene encoding the C-terminal domain of σ^A^ containing the last 112 residues (aa 417-528), denoted σ^A^_C112_) was amplified from pCDF-1b-6HisMtbσ^A^_C170_, fused to the β‐flap‐tip helix (aa 815-829) with an artificial linker (GSSGSG), and inserted into the NcoI/XhoI site of pET28b to express His_6_-σ^A^_C112_-β_tip._ It was co-expressed with either the wildtype or mutant WhiB3 for the EMSA experiment; Kanamycin (Kan^+^) | (2) |
| pET28b-6HisMtbσ^A^_C82_-β_tip_ | A modification of the pET28b-6HisMtbσ^A^_C112_-β_tip_ plasmid by site-directed mutagenesis to express the chimera protein with a shorter C-terminal σ^A^_C82_ containing the last 82 residues of σ^A^ (aa 446- 528 aa). It was co-expressed with either the full-length or the truncated WhiB3 for crystallization; Kan^+^ | (2) |
| pET21-MtbWhiB3 | The full-length *Mtb* *whiB3* gene was amplified from *Mtb* H37Rv genomic DNA and inserted into the NdeI/XhoI site of pET21 for expressing tagless Mtb WhiB3; Ampicillin (Amp^+^) | This study |
| pET21b-MtbWhiB3TR | A modification of pET21b-MtbWhiB3 by site-directed mutagenesis to express a truncated Mtb WhiB3 (aa 1-90) without the last ten residues in the C-terminus. It was co-expressed either with pET28b-6HisMtbσ^A^_C112_-β_tip_ for the EMSA experiment, or with pET28b-6HisMtbσ^A^_C82_-β_tip_ for crystallization; Amp^+^ | This study |
| pET21-MtbWhiB3 mutant (W15A, W17A, W15AW17A, F31A, F32A, E71A, W76A, R38A, R40A, R42A) | A modification of pET21-MtbWhiB3 by site-directed mutagenesis for expressing the tagless WhiB3 carrying mutation of either W15A, W17A, W15AW17A, F31A, F32A, E71A, W76A, R38A, R40A, R42A; Amp^+^ | This study |
| pET21-WhiB3FL-3RtoA | A modification of pET21-MtbWhiB3 by site-directed mutagenesis for expressing the tagless WhiB3 carrying triple mutations of R38A, R40A and R42A; Amp^+^ | This study |
| Plasmids for creating the *∆whiB3* mutant in *Msm* and complementation by *Msm* WhiB3 | | |
| pJV53-GFP | A gift from Dr. Yicheng Sun’s group for expressing recombination proteins gp60 and gp61 of mycobacteriophage Che9c and a GFP reporter. This plasmid was used for the unmarked deletion of *whiB3* in *Msm*; Kan^R^ | (Mao et al., 2016) |
| pUC-Hyg | A gift plasmid from Dr. Yicheng Sun’s group contains a *dif*-ﬂanked hygromycin resistance cassette. It was used for constructing DNA fragment the unmarked deletion of *whiB7* in *Msm*; Amp^R^, Hyg^R^ | (Mao et al., 2016) |
| pKW08-Lx-Int | An integration plasmid for gene expression in *Msm* under the inducible *tetR* promoter in mycobacteria, containing *LuxAB* genes, L5 integrase gene and phage attachment *attP* sequences; Hyg^R^ | (Williams et al., 2010) |
| pUC-Hyg+MsmwhiB3LR | A 579-bp and a 720-bp DNA fragments from the upstream and downstream, respectively, of the *Msm* *whiB3* gene were amplified from *Msm* MC^^2^ 155 genomic DNA and cloned into the left arm (KpnI-BglII site) and right arm (Nco-SpeI site) flanking the hygromycin resistance cassette in pUC-Hyg for the unmarked *whiB3* deletion; Amp^R^, Hyg^R^ | This study |
| pKW08-Int-PwhiB3-msmWhiB3 | The DNA fragment encoding WhiB3 and the 592-bp upstream DNA inserted into the XhoI/HindIII site of pKW08-Lx-Int plasmid to express the wild-type WhiB3 under the native promoter. This plasmid was used as a positive control role of the C-terminal arginine-rich WhiB3 in WhiB3-dependent transcriptional regulation by RT-qPCR; Hyg^R^ | This study |
| pKW08-Int-PwhiB3-msmWhiB3TR | This plasmid was modified from pKW08-Int-PwhiB3-msmWhiB3 by site-directed mutagenesis at Genscript to express the C-terminal truncated WhiB3 (aa 1-89, corresponding to aa 1-89 of *Mtb* WhB3) under the native promoter. This plasmid was used to test the role of the C-terminal Arg-rich WhiB3 in the WhiB3-dependent transcriptional regulation by RT-qPCR; Hyg^R^ | This study |
| pKW08-Int-PwhiB3-msmWhiB3-3RtoA | This plasmid was modified from pKW08-Int-PwhiB3-msmWhiB3 by site-directed mutagenesis at Genscript to the tagless WhiB3 carrying triple mutations of R38A, R40A and R42A under the native promoter. This plasmid was used to test the role of the conserved Arg-rich motif (containing R38, R40 and R42) in the WhiB3-dependent transcriptional regulation by RT-qPCR; Hyg^R^ | This study |

**Supplementary Table S2.** Comparison of the polar contacts between the protein and nucleotides in the WhiB3:σ^A^_4_-β_tip_:*P_whiB7_* and WhiB7:σ^A^_4_-β_tip_:*P_whiB7_* complexes, as shown in Figure S7.

| **WhiB3:σ^A^_4_-β_tip_:*P_whiB7_*** | | | **WhiB7:σ^A^_4_-β_tip_:*P_whiB7_*** | | |
| --- | --- | --- | --- | --- | --- |
| **Protein** | **DNA** | **Distance (Å)*** | **Protein** | **DNA** | **Distance (Å)*** |
| WhiB3:G36:N | D:A39:OP1 | 2.77 | WhiB7:R81:NH2 | C:A40:O3' | 2.84 |
| WhiB3:R38:NH1 | D:C36:O2 | 3.10 |  | C:T39:OP1 | 2.84 |
|  | D:C37:O4' | 2.83 | WhiB7:R83:O | C:T39:O4' | 2.92 |
| WhiB3:R38:NH2 | D:C37:O2 | 2.82 | WhiB7:R83:NH1 | C:T39:O2 | 3.40 |
|  | D:G38:O4' | 3.20 |  | D:T41:O4' | 3.11 |
| σ_A_^4^:R470:NH1 | D:T40:OP2 | 2.90 | WhiB7:R83:NH2 | C:T39:O2 | 2.76 |
| σ_A_^4^:R470:NH2 | D:T40:OP2 | 2.82 |  | C:C38:O4' | 3.04 |
| σ_A_^4^:T499:OG1 | D:T41:OP1 | 2.72 | WhiB7:R85:N | D:T42:O2 | 3.34 |
| σ_A_^4^:T499:N | D:T41:OP1 | 2.79 | WhiB7:R87:NE | D:T43:OP1 | 2.96 |
| σ_A_^4^:E501:OE1 | C:A43:N6 | 3.32 | WhiB7:R87:NH1 |  | 3.13 |
| σ_A_^4^:E501:OE2 | C:A43:N7 | 3.36 | σ_A_^4^:R470:NH1 | C:G36:OP1 | 3.10 |
| σ_A_^4^:R502:NE | D:T40:OP2 | 3.03 | σ_A_^4^:R470:NH2 | C:G36:OP2 | 2.35 |
| σ_A_^4^:R502:NH2 | D:T40:OP2 | 3.05 | σ_A_^4^:R478:NH1 | D:C31:OP1 | 3.36 |
| σ_A_^4^:R504:NH1 | C:G44:O6 | 2.44 | σ_A_^4^:R478:NH2 |  | 2.60 |
| σ_A_^4^:R504:NH2 | C:G44:N7 | 2.69 | σ_A_^4^:T488:OG1 | D:C31:OP2 | 2.60 |
| σ_A_^4^:Q505:NE2 | D:T41:O4 | 3.28 | σ_A_^4^:L489:N |  | 3.12 |
|  |  |  | σ_A_^4^:T499:OG1 | C:T35:OP2 | 3.16 |
|  |  |  | σ_A_^4^:T499:N |  | 2.78 |
|  |  |  | σ_A_^4^:E501:OE2 | D:C33:N4 | 2.98 |
|  |  |  | σ_A_^4^:R502:NE | C:G36:OP2 | 2.61 |
|  |  |  | σ_A_^4^:R502:NH2 |  | 3.10 |
|  |  |  | σ_A_^4^:R504:NH2 | D:C33:C5 | 3.33 |
|  |  |  | σ_A_^4^:Q505:NE2 | C:T35:O4 | 2.90 |

Note: Only polar contacts shown in Fig. S7 are listed in the table for comparison. A 3.4 Å cutoff was used for polar contacts. In addition, N2 of G36 is close to R38 (3.1 and 3.2 Å away from NH1 and CZ, respectively) and could potentially form a weak N-H/π hydrogen bond with R38. However, the contribution of this interaction needs to be validated. Nucleotide numbering of the C chain is based on the position of the -35 element, as shown in Fig. S7. Numbering of the nucleotides in the D chain is based on the paired base in the C chain for easy comparison between Table S2 and Fig. S7.

**Supplementary Figures**

**Supplementary Figure S1. Sequence alignments of *Mtb* Wbl proteins.** A, Multiple protein sequence alignment of seven *Mtb* Wbl proteins. Highlighted are the four conserved cysteines in the [4Fe-4S] cluster binding motif and the β turn shared by all Wbl proteins, together with the subclass-specific DNA binding motifs (the Arg-rich motif in WhiB3 and the AT-hook in WhiB7). Note that *Mtb* WhiB5 has a distinct pattern (Cx_3_C) in the Fe-S cluster binding motif between the second and the third conserved Cys instead of a Cx_2_C pattern shared by all other *Mtb* Wbl members. The N- and C-terminal regions referred to in this study are highlighted. B, the protein sequence of *Mtb* WhiB3, followed by the sequence logo of the representative WhiB3-subclass members using WebLogo (3). For generating the WhiB3 subclass sequence logo, *Mtb* WhiB3 is aligned against 49 orthologs in Actinobacteria, including *Streptomycineae, S. avermitilis, S. viridochromogenes, S. scabiei, S. griseoflavus, S. venezuelae, S. griseus, S. hygroscopicus, S. pristinaespiralis, S. albus, S. clavuligerus; Catenulispora acidiphila; Micromonospora, M. sp. ATCC39149, M. aurantiaca; Saccharomonospora viridis; Saccharopolyspora erythraea; Amycolatopsis mediterranei U32; Actinosynnema mirum DSM43827; Thermobifida fusca YX; Nocardiopsis dassonvillei subsp. dassonville DSM43111; Geodermatophilus obscurus DSM 43160; Nakamurella multipartite; Gordonia bronchialis; Nocardia farcinica IFM 10152; Segniliparus rotundus; Tsukamurella paurometabola; Rhodococcus, R. opacus, R. jostii, R. erythropolis; Mycobacterium, M. ulcerans, M. sp. MCS, M. sp. JLS, M. smegmatis, M. leprae, M. abscessus subsp. abscessus, M. avium, M. bovis; Corynebacterium, C. pseudotuberculosis, C. kroppenstedtii DSM 44385, C. jeikeium, C. glutamicum, C. efficiens, C. aurimucosum; Kribbella flavida; Kineococcus radiotolerans; Beutenbergia cavernae; Cellulomonas flavigena; Kytococcus sedentarius; Intrasporangium calvum; Jonesia denitrificans*. The residues in *Mtb* WhiB3 that are invariant or highly conserved (varied residues with a similar polarity) in the WhiB3 subclasses are highlighted in red and blue, respectively. The three Arg residues (R38, R40 and R42 in *Mtb*) in involved in DNA binding are highlighted in the sequence logo. As demonstrated in the sequence logo, R38 and R42 of Mtb WhiB3 are invariant in the WhiB3 subclass, while R40 is a variant.


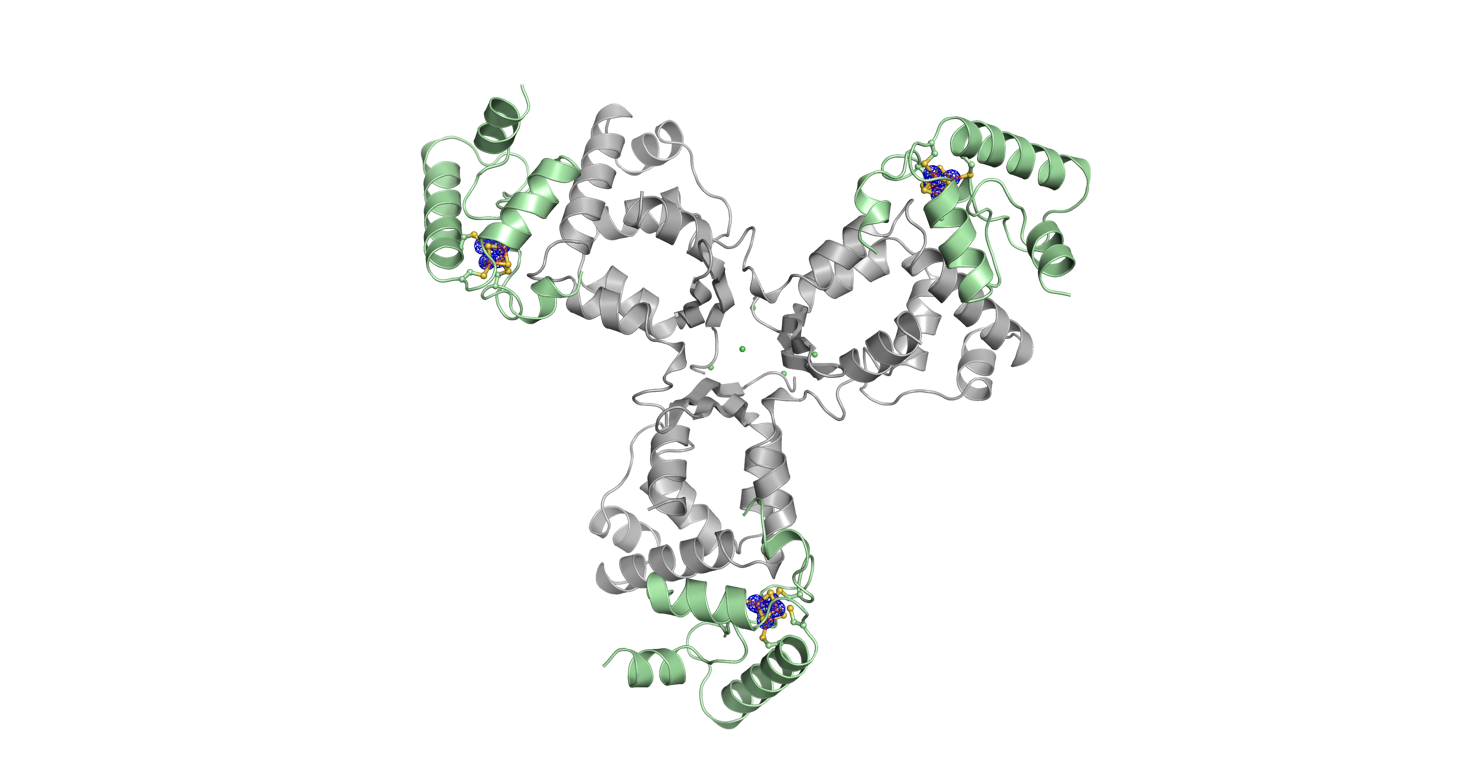

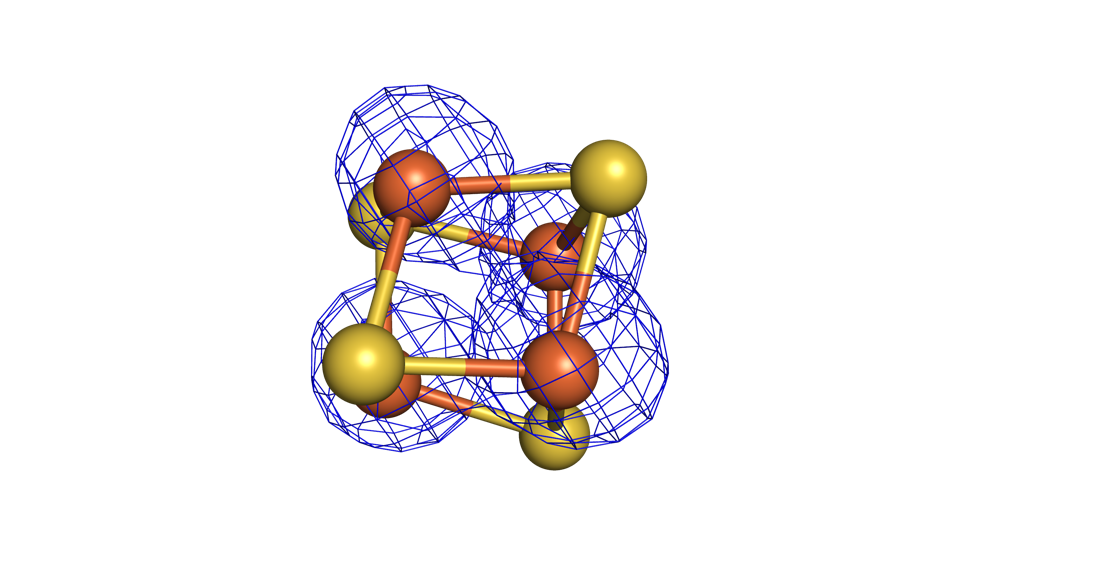


**Supplementary Figure S2. Anomalous density map of the WhiB3TR:σ^A^_4-_β_-tip_ in the P4_3_2_1_2 form**. The anomalous density map is highlighted in blue and contoured at 15 σ. The cartoon representation of WhiB3 and σ^A^_4-_β_-tip_ are highlighted in pale green and gray, respectively. The Fe, Ni and S atoms are shown in spheres, with Fe colored in orange, Ni in pale green, and S in yellow. The anomalous diffraction data were collected at 7200 eV. The statistics for the phasing data are summarized in Table 1 and the phasing FOM is 0.58.


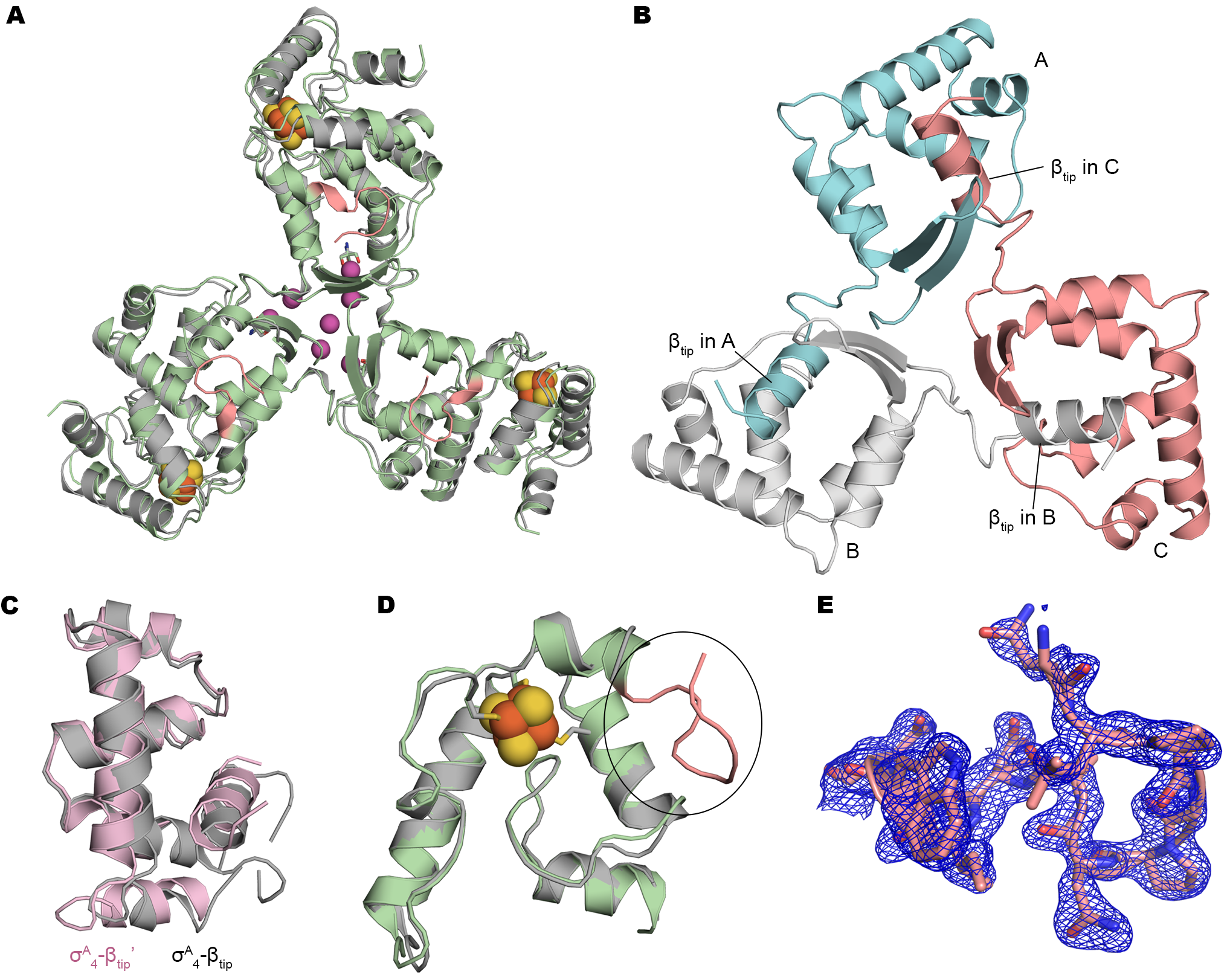


**Supplementary Figure S3. Comparison of the WhiB3TR:σ^A^_4-_β_-tip_ structures in the P4_3_2_1_2 and R3 forms.** A, an overlay of the trimeric structure in P43212 (grey) and R3 (green), with an average Cα root-mean-square deviation (RMSD_Cα_) of 1.55 Å among the 546 aligned Cα atoms. The N-terminal residues of WhiB3 in the R3 trimer are highlighted in salmon pink. B, the intermolecular interactions between σ^A^_4_ and β_tip_^ʹ^ in the trimer of the WhiB3TR:σ^A^_4-_β_tip_ complexes. β_tip_ in one WhiB3TR:σ^A^_4_-β_tip_ complex is extended to the neighboring complex and forms the interactions with σ^A^_4_ in the second complex, resembling the interactions between σ^A^_4_ and β_tip_ in the WhiB7:σ^A^_4_-β_tip_ complex as demonstrated in Panel C_._ The three copies (labeled as A, B and C) of σ^A^_4_-β_tip_ in the trimer are colored grey, pale green and salmon pink, respectively. WhiB3 is not shown in the trimer structure for clarity. C, an overlay of the σ^A^_4_-β_tip_^ʹ^ in the R3 structure with σ^A^_4_-β_tip_ in the WhiB7:σ^A^_4_-β_tip_ complex (PDB:7KUG), with an average RMSD_Cα_ of 0.76 Å among 57 aligned Cα atoms in σ^A^ (447-528). D, an overlay of WhiB3 in the P4_3_2_1_2 (grey) and R3 (pale green, except for the N-terminal residues highlighted in salmon pink) structures. E, 2Fo-Fc density map of the N-terminal residues (aa 6-16) in the R3 structure, contoured at 1.0 σ.

**Supplementary Figure S4. UV-Visible spectra of the samples from the co-expression and affinity purification of His6-σ^A^_C170_ with the tagless WhiB3 proteins (wildtype and mutants as indicated).** σ^A^_C170_ contains the last 170 residues in the C-terminus of σ^A^ (see Table S1). The intensity of the absorption peak around 410 nm is indicative of the occupancy of the [4Fe-4S] cluster in the σ^A^_C170_-bound WhiB3 of the purified samples. The absorption spectra were normalized at 280 nm based on the protein concentrations estimated by the Pierce Bradford Assay Kit (Thermo Fisher Scientific).


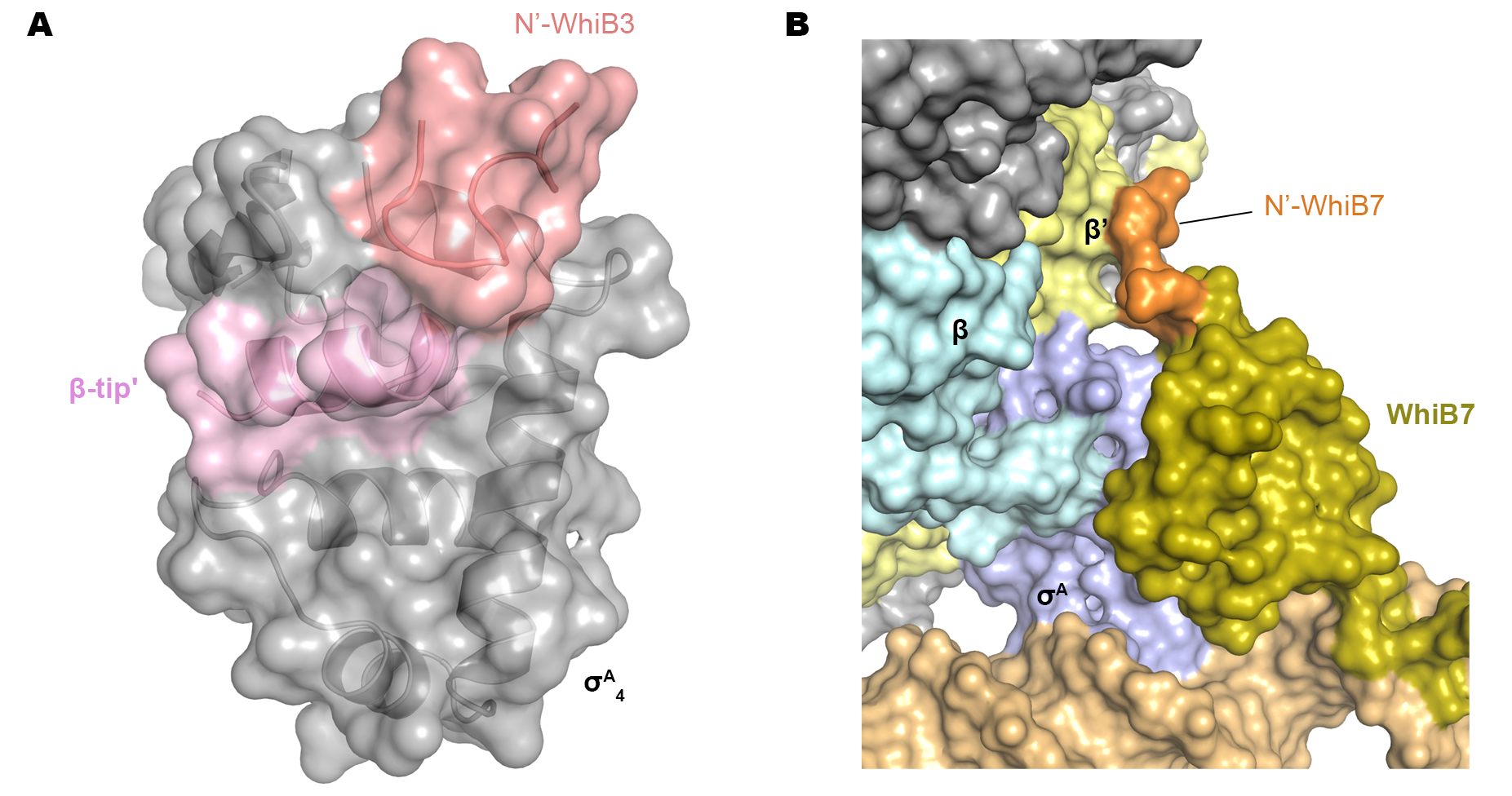


**Supplementary Figure S5.** Interactions between the N-terminus WhiB3 with β_-tip_ compared with WhiB7. A, surface representation of the interactions between the N-terminal WhiB3 residues (aa 6-16, in salmon pink) with σ^A^_4_ (grey) and β_tip_^ʹ^ (pink). The buried interface surface between N-terminal WhiB3 residues and σ^A^_4_-β_tip_^ʹ^ is estimated at 370.1 A^2^ using the online macromolecular interface tool PISA (4). The β_tip_ from the neighboring complex was shown (labeled as β_tip_^ʹ^) to reflect the physiologically relevant contacts between the two subunits in the RNAP holoenzyme. B, a close-up view of the boxed region of the WhiB7-RNAP-DNA complex in Fig. 3B. The N terminus of WhiB7 (brown) sticks into the RNA polymerase in the opposite direction relative to the N-terminal WhiB3 and interacts with the β^ʹ^-subunit of RNAP.

**Figure S6**. EMSAs of the WhiB3:σ^A^_4_-β_tip_ complex with A) the predicted *pks3* promoter DNA (*P_pks3_*), B) the predicted *pks2* promoter DNA (*P_pks2_*), and C) *P_whiB7_*. The -35 hexamers in the *pks2* and *pks3* promoter were predicted based on the consensus sequences but have not been experimentally confirmed. **Supplementary Figure S7.** Analysis of the protein–DNA contacts in the WhiB3:σ^A^_4_-β_tip_:*P_whiB7_* and WhiB7:σ^A^_4_-β_tip_:*P_whiB7_* complexes_._ A break of base-base stacking of *P_whiB7_* in the WhiB7:σ^A^_4_-β_tip_:*P_whiB7_* complex is highlighted by a red circle, correlated with the starting point of DNA bending caused by WhiB7 binding and in contrast to the corresponding nucleotides in WhiB3:σ^A^_4_-β_tip_:*P_whiB7_* complex (in blue circle). Nucleotide numbering is based on the position of the -35 element. The figure was generated by the online server DNAproDB. A maximum distance of 3.4 Å was applied for calculating protein–DNA contacts. The functional groups of the nucleotides (N=A, T, G, C) in contact with a protein are highlighted by the color using the scheme shown in the inset on the right: sugars in yellow, phosphates in orange, and bases in gray. Contacts in the minor grooves are highlighted in pink, and the major grooves in cyan. The residues in contact with DNA are highlighted by the colored shape based on the secondary structure of the peptide where they are in: helices are highlighted by red dots, β strands by green triangles, and loops by blue squares. The phosphodiester bond between the neighboring nucleotide is shown as an orange line, while the base-base stacking is shown by a gray line. Watson-Crick base pairs (W.C. BP) are shown in black lines, Hoogsteen base pairs (Hoog. BP) shown in red lines, while others in red dash lines. The polar contacts between the protein complex and DNA are listed in Table S2.


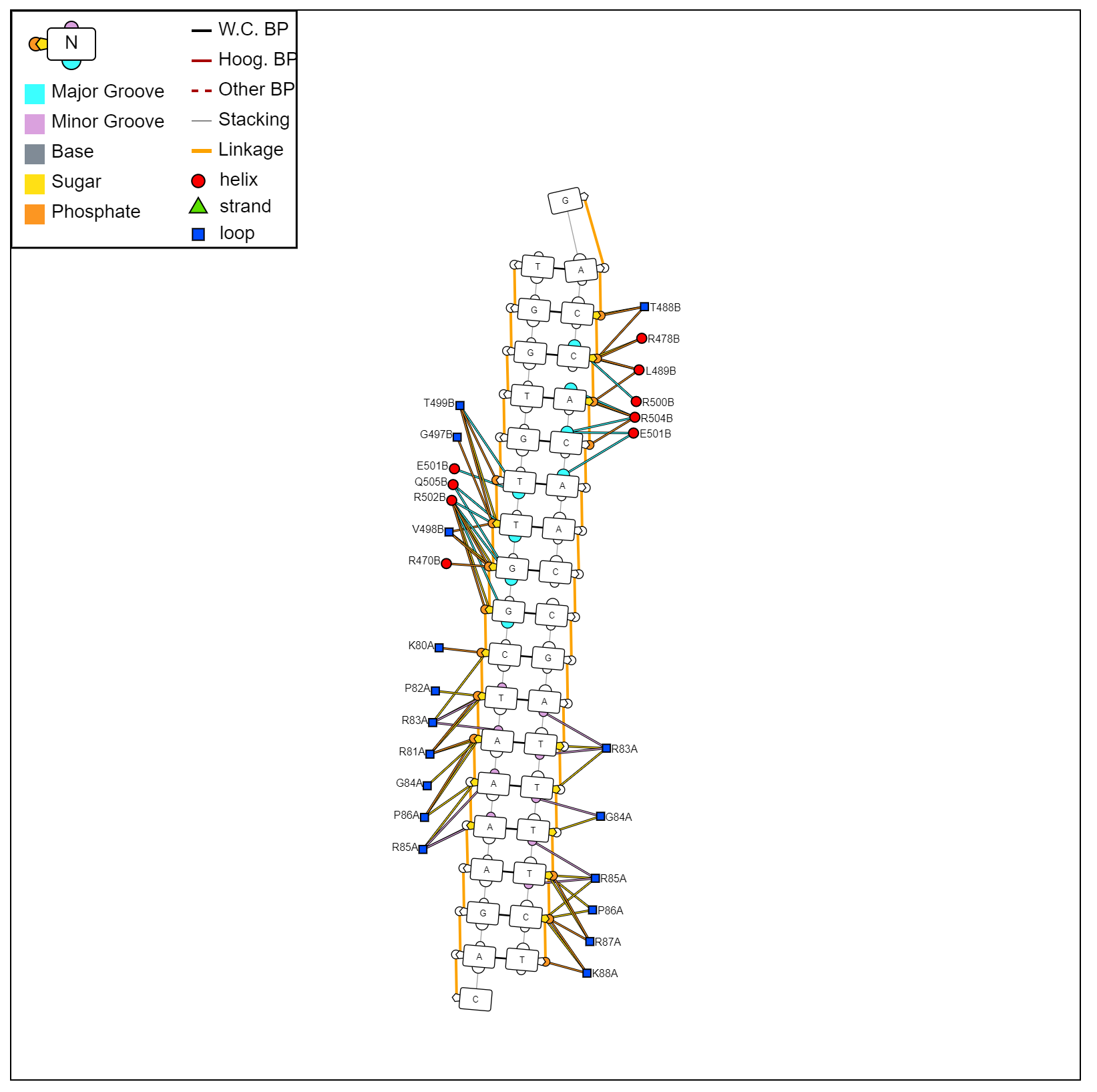


WhiB3:σ^A^_4_-β_tip_:*P_whiB7_*

WhiB7:σ^A^_4_-β_tip_:*P_whiB7_*


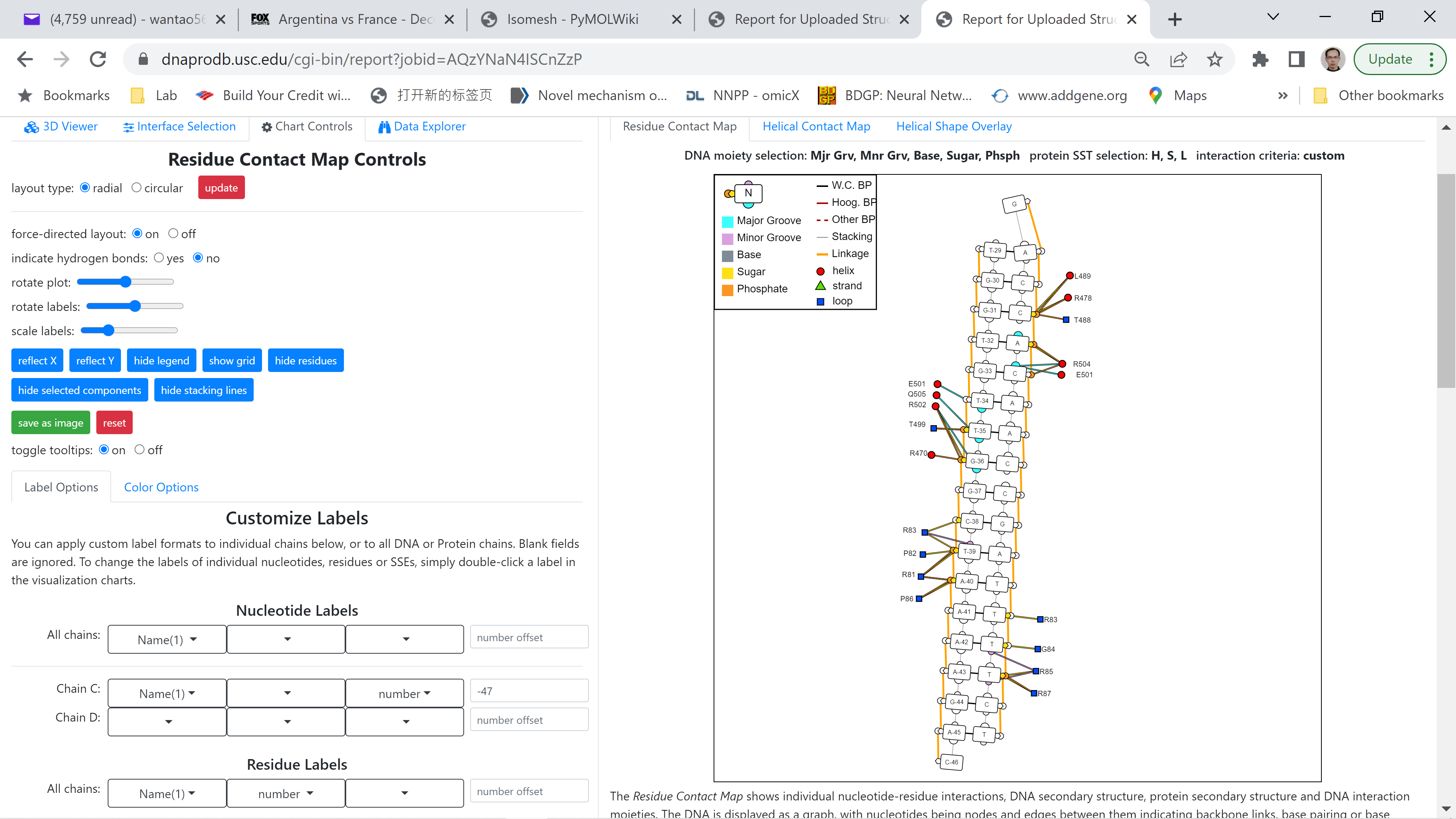

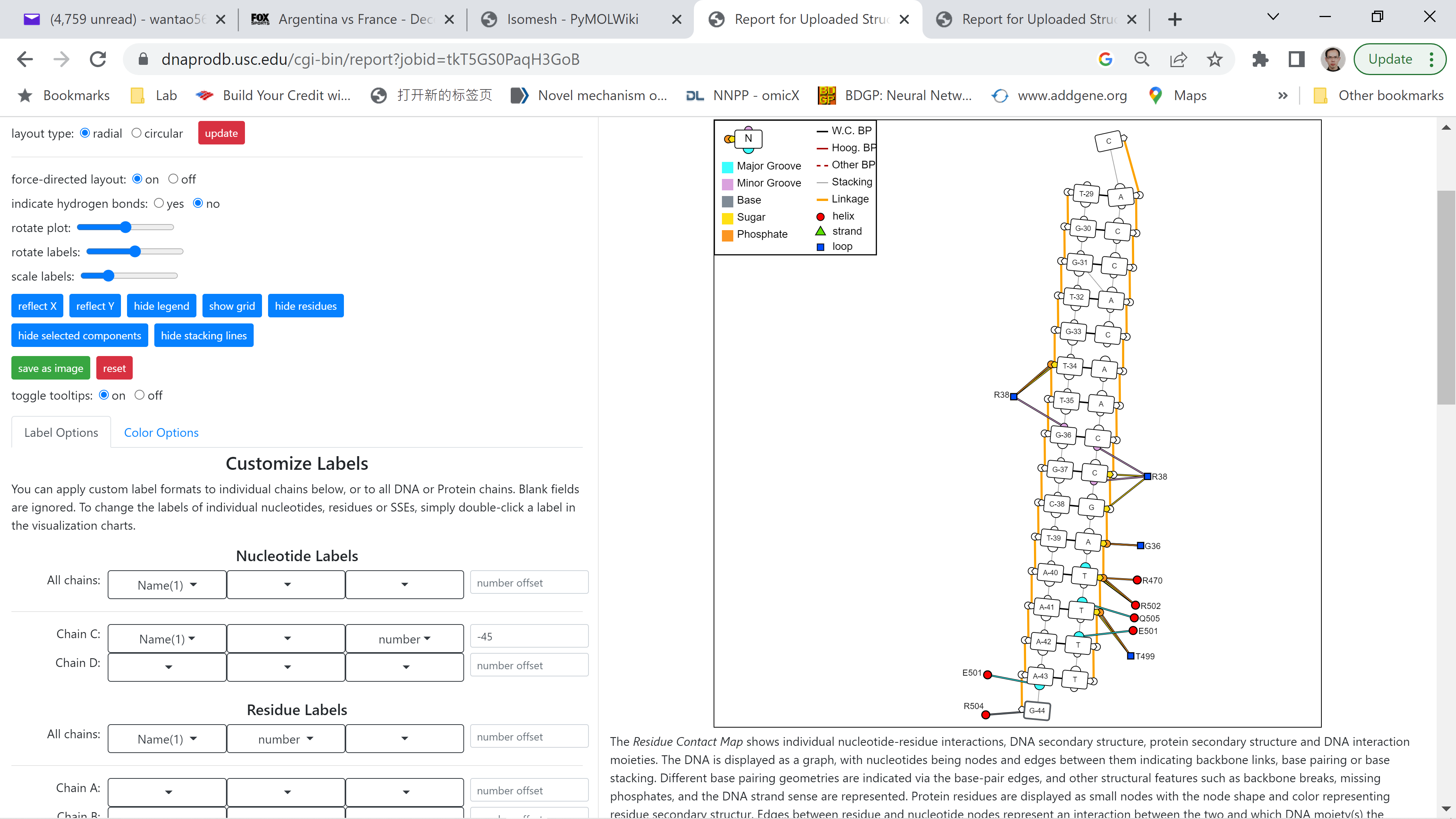

**Figure S8.** Comparison of the *P_whiB7_* DNA structure in the WhiB3:σ^A^_4_-β_tip_:*P_whiB7_* and WhiB7:σ^A^_4_-β_tip_:*P_whiB7_* complexes. A) Overlay of *P_whiB7_* in the two Wbl:σ^A^_4_-β_tip_:*P_whiB7_* complexes. The *P_whiB7_* DNA in WhiB7:σ^A^_4_-β_tip_:*P_whiB7_* is colored gray with the exception for the AT-rich region highlighted in blue. *P_whiB7_* in WhiB3:σ^A^_4_-β_tip_:*P_whiB7_* is colored orange. The residues in WhiB3 (R38) and WhiB7 (AT-hook) that interact with *P_whiB7_* are highlighted as indicated. The minor groove around the AT-rich region of *P_whiB7_* in the WhiB3:σ^A^_4_-β_tip_:*P_whiB7_* complex is highlighted in the red circle. B) Analysis of the minor- and major-groove width of *P_whiB7_* in WhiB3:σ^A^_4_-β_tip_:*P_whiB7_* (B3 minor and B3 major) and WhiB7:σ^A^_4_-β_tip_:*P_whiB7_* (B7 minor and B7 major), respectively. The groove width is defined as the distance between the closest phosphates subtracted by 5.8 Å (the sum of the van der Waals radii of the two phosphate atoms). The dashed line indicates the mean value of canonical minor groove widths in B-form DNAs. Nucleotide numbering is based on the position of -35 element. All statistics were based on output of the Web 3DNA 2.0 server (<http://web.x3dna.org/>). Note the *P_whiB7_* DNA used for the WhiB3:σ^A^_4_-β_tip_:*P_whiB7_* crystal is 2-bp shorter than that used in the WhiB7:σ^A^_4_-β_tip_:*P_whiB7_* crystal.

**References**

1. Wan, T., Li, S., Beltran, D. G., Schacht, A., Zhang, L., Becker, D. F., and Zhang, L. (2020) Structural basis of non-canonical transcriptional regulation by the σ^A^-bound iron-sulfur protein WhiB1 in *M. tuberculosis*. *Nucleic Acids Res* **48**, 501-516

2. Wan, T., Horova, M., Beltran, D. G., Li, S., Wong, H. X., and Zhang, L. M. (2021) Structural insights into the functional divergence of WhiB-like proteins in Mycobacterium tuberculosis. *Mol Cell* **81**, 2887-2900 e2885

3. Crooks, G. E., Hon, G., Chandonia, J. M., and Brenner, S. E. (2004) WebLogo: a sequence logo generator. *Genome Res* **14**, 1188-1190

4. Krissinel, E., and Henrick, K. (2007) Inference of macromolecular assemblies from crystalline state. *J Mol Biol* **372**, 774-797
